# Genome-wide association study of lifetime cannabis use based on a large meta-analytic sample of 32 330 subjects from the International Cannabis Consortium

**DOI:** 10.1038/tp.2016.36

**Published:** 2016-03-29

**Authors:** S Stringer, C C Minică, K J H Verweij, H Mbarek, M Bernard, J Derringer, K R van Eijk, J D Isen, A Loukola, D F Maciejewski, E Mihailov, P J van der Most, C Sánchez-Mora, L Roos, R Sherva, R Walters, J J Ware, A Abdellaoui, T B Bigdeli, S J T Branje, S A Brown, M Bruinenberg, M Casas, T Esko, I Garcia-Martinez, S D Gordon, J M Harris, C A Hartman, A K Henders, A C Heath, I B Hickie, M Hickman, C J Hopfer, J J Hottenga, A C Huizink, D E Irons, R S Kahn, T Korhonen, H R Kranzler, K Krauter, P A C van Lier, G H Lubke, P A F Madden, R Mägi, M K McGue, S E Medland, W H J Meeus, M B Miller, G W Montgomery, M G Nivard, I M Nolte, A J Oldehinkel, Z Pausova, B Qaiser, L Quaye, J A Ramos-Quiroga, V Richarte, R J Rose, J Shin, M C Stallings, A I Stiby, T L Wall, M J Wright, H M Koot, T Paus, J K Hewitt, M Ribasés, J Kaprio, M P Boks, H Snieder, T Spector, M R Munafò, A Metspalu, J Gelernter, D I Boomsma, W G Iacono, N G Martin, N A Gillespie, E M Derks, J M Vink

**Affiliations:** 1 Department of Complex Trait Genetics, VU Amsterdam, Center for Neurogenomics and Cognitive Research, Amsterdam, The Netherlands; 2 Department of Psychiatry, Academic Medical Centre, Amsterdam, The Netherlands; 3 Department of Biological Psychology/Netherlands Twin Register, VU University, Amsterdam, The Netherlands; 4 Neuroscience Campus Amsterdam, Amsterdam, The Netherlands; 5 Department of Developmental Psychology and EMGO Institute for Health and Care Research, VU University, Amsterdam, The Netherlands; 6 The Hospital for Sick Children Research Institute, Toronto, Canada; 7 Department of Psychology, University of Illinois Urbana-Champaign, Champaign, IL, USA; 8 Department of Human Neurogenetics, Brain Center Rudolf Magnus, University Medical Center Utrecht, Utrecht, The Netherlands; 9 Department of Psychology, University of Minnesota, Minneapolis, MN, USA; 10 Department of Public Health, Hjelt Institute, University of Helsinki, Helsinki, Finland; 11 Estonian Genome Center, University of Tartu, Tartu, Estonia; 12 Department of Epidemiology, University Medical Center Groningen, University of Groningen, Groningen, The Netherlands; 13 Psychiatric Genetics Unit, Vall d'Hebron Research Institute (VHIR), Universitat Autònoma de Barcelona, Barcelona, Spain; 14 Department of Psychiatry, Hospital Universitari Vall d'Hebron, Barcelona, Spain; 15 Biomedical Network Research Centre on Mental Health (CIBERSAM), Barcelona, Spain; 16 Twin Research and Genetic Epidemiology, King's College London, London, UK; 17 Biomedical Genetics Department, Boston University School of Medicine, Boston, MA, USA; 18 Analytic and Translational Genetics Unit, Massachusetts General Hospital, Boston, MA, USA; 19 Stanley Center for Psychiatric Research, Broad Institute of MIT and Harvard, Cambridge, MA, USA; 20 Department of Medicine, Harvard Medical School, Boston, MA, USA; 21 School of Social and Community Medicine, University of Bristol, Bristol, UK; 22 MRC Integrative Epidemiology Unit (IEU), University of Bristol, Bristol, UK; 23 Department of Psychiatry, Virginia Institute for Psychiatric and Behavior Genetics, Virginia Commonwealth University, Richmond, VA, USA; 24 Research Centre Adolescent Development, Utrecht University, Utrecht, The Netherlands; 25 Department of Psychology and Psychiatry, University of California San Diego, La Jolla, CA, USA; 26 The LifeLines Cohort Study, University of Groningen, Groningen, The Netherlands; 27 Department of Psychiatry and Legal Medicine, Universitat Autònoma de Barcelona, Barcelona, Spain; 28 Genetic Epidemiology, Molecular Epidemiology and Neurogenetics Laboratories, QIMR Berghofer Medical Research Institute, Brisbane, QLD, Australia; 29 Department of Psychiatry, University Medical Center Groningen, University of Groningen, Groningen, The Netherlands; 30 Department of Psychiatry, Washington University School of Medicine, St Louis, MO, USA; 31 Brain and Mind Research Institute, University of Sydney, Sydney, NSW, Australia; 32 Department of Psychiatry, University of Colorado Denver, Aurora, CO, USA; 33 Institute of Public Health and Clinical Nutrition, University of Eastern Finland, Kuopio, Finland; 34 Department of Mental Health and Substance Abuse Services, National Institute for Health and Welfare, Helsinki, Finland; 35 Department of Psychiatry, University of Pennsylvania Perelman School of Medicine, Philadelphia, PA, USA; 36 Department of Molecular, Cellular and Developmental Biology, University of Colorado Boulder, Boulder, CO, USA; 37 Department of Psychology, University of Notre Dame, Notre Dame, IN, USA; 38 Developmental Psychology, Tilburg University, Tilburg, The Netherlands; 39 Interdisciplinary Center for Pathology and Emotion Regulation, University Medical Center Groningen, University of Groningen, Groningen, The Netherlands; 40 Department of Physiology and Nutritional Sciences, University of Toronto, Toronto, ON, Canada; 41 Department of Psychological and Brain Sciences, Indiana University Bloomington, Bloomington, IN, USA; 42 Department of Psychology and Neuroscience, Institute for Behavioral Genetics, University of Colorado Boulder, Boulder, CO, USA; 43 Department of Psychiatry, University of California San Diego, La Jolla, CA, USA; 44 Rotman Research Institute, Baycrest, Toronto, ON, Canada; 45 Department of Psychology and Psychiatry, University of Toronto, Toronto, ON, Canada; 46 Center for the Developing Brain, Child Mind Institute, New York, NY, USA; 47 Institute for Molecular Medicine Finland, University of Helsinki, Helsinki, Finland; 48 UK Centre for Tobacco and Alcohol Studies and School of Experimental Psychology, University of Bristol, Bristol, UK; 49 Department of Psychiatry, Genetics, and Neurobiology, Yale University School of Medicine and VA CT, West Haven, CT, USA; 50 Behavioural Science Institute, Radboud University, Nijmegen, The Netherlands

## Abstract

Cannabis is the most widely produced and consumed illicit psychoactive substance worldwide. Occasional cannabis use can progress to frequent use, abuse and dependence with all known adverse physical, psychological and social consequences. Individual differences in cannabis initiation are heritable (40–48%). The International Cannabis Consortium was established with the aim to identify genetic risk variants of cannabis use. We conducted a meta-analysis of genome-wide association data of 13 cohorts (*N*=32 330) and four replication samples (*N*=5627). In addition, we performed a gene-based test of association, estimated single-nucleotide polymorphism (SNP)-based heritability and explored the genetic correlation between lifetime cannabis use and cigarette use using LD score regression. No individual SNPs reached genome-wide significance. Nonetheless, gene-based tests identified four genes significantly associated with lifetime cannabis use: *NCAM1*, *CADM2*, *SCOC* and *KCNT2*. Previous studies reported associations of *NCAM1* with cigarette smoking and other substance use, and those of *CADM2* with body mass index, processing speed and autism disorders, which are phenotypes previously reported to be associated with cannabis use. Furthermore, we showed that, combined across the genome, all common SNPs explained 13–20% (*P*<0.001) of the liability of lifetime cannabis use. Finally, there was a strong genetic correlation (*r*_g_=0.83; *P*=1.85 × 10^−8^) between lifetime cannabis use and lifetime cigarette smoking implying that the SNP effect sizes of the two traits are highly correlated. This is the largest meta-analysis of cannabis GWA studies to date, revealing important new insights into the genetic pathways of lifetime cannabis use. Future functional studies should explore the impact of the identified genes on the biological mechanisms of cannabis use.

## Introduction

Cannabis is the most widely produced and consumed illicit psychoactive substance worldwide.^1^ Following initiation, occasional cannabis use can progress to frequent use, abuse and dependence. About 1 in 10 occasional users becomes dependent, which is associated with physical, psychological, social and occupational consequences.^2, 3^ Despite the increasing use of cannabis for medicinal purposes,^4^ associations with adverse health effects have been reported.^5, 6^ These include increased risk for psychiatric outcomes, including psychosis, schizophrenia, schizotypal personality disorder and mania.^7, 8^ Early cannabis use appears to moderate relationship between polygenic risk scores for schizophrenia and brain maturation.^9^ In view of expanding medicalization and decriminalization, the potential consequences, and the debate surrounding the benefits versus adverse consequences associated with cannabis use,^10^ understanding the genetics of cannabis use should be a public health priority.^11^

The risk of lifetime cannabis use, defined as any use of cannabis during the lifetime, varies between individuals. Previous studies have shown that individual differences in lifetime cannabis use can be partly explained by genetic differences between individuals; a meta-analysis of twin studies reported significant heritability estimates of lifetime cannabis use of 48% for males and 40% for females.^12^ Shared environmental factors, such as cannabis availability and parental monitoring,^13, 14^ also have a role accounting for 25 and 39% of the risk for males and females, respectively.^12^ Also, there is substantial overlap in the genetic risks underlying lifetime cannabis use and cannabis use disorder.^15^

Several studies have sought to identify specific genetic risk factors associated with cannabis use phenotypes. Genome-wide linkage studies have revealed suggestive evidence for linkage across many chromosomes.^16, 17, 18, 19, 20^ With very little consistency across studies, nearly all findings failed to meet genome-wide significance. The one study examining lifetime cannabis use^16^ reported a nonsignificant linkage locus on chromosome 18 (LOD score=1.97).

Candidate gene studies, including reports examining the *CNR1*, *GABRA2*, *FAAH* and *ABCB1* genes have detected some significant associations with cannabis use but again, replication has been inconsistent.^21, 22, 23^ On the basis of a sample of 7452 Caucasian individuals, Verweij *et al.*^21^ found no gene-based associations between the frequency of cannabis use and 10 candidate genes identified by Agrawal and Lynskey.^24^ Overall, the results of candidate-gene studies are inconclusive; some associations have been replicated a few times, but failed to replicate in other studies. Moreover, findings may be further distorted due to publication bias favouring significant results.

As an alternative to the candidate-gene approach, the genome-wide association study (GWAS) is a hypothesis-free method that aims to detect novel genetic variants involved in complex traits. To date, three GWASs of cannabis use phenotypes have been published: one GWAS of cannabis dependence in 708 cannabis-dependent individuals and 2346 controls;^25^ a GWAS meta-analysis of lifetime cannabis use based on two studies with a combined sample size of 10 091 individuals (40.7% users);^26^ and a recent GWAS of lifetime cannabis use and age of cannabis use onset based on a sample of 6744 individuals (of whom 20% were users).^27^ None of the studies identified any genome-wide significant associations. This was likely due to the small effect sizes typical of common variants underpinning highly polygenic traits,^28^ thereby indicating a need for larger sample sizes. In this context, the success of larger GWASs and international consortia examining a variety of complex traits is encouraging.^29^ For example, multiple large meta-analyses of GWA results for number of cigarettes smoked per day have independently identified associations on chromosome 15q25 spanning the α5, α3 and β4 nicotinic receptor subunit gene clusters (*CHRNA5*, *CHRNA3*, *CHRNB4*).^30, 31, 32^

These and other recent GWA findings^29^ clearly illustrate the need for larger sample sizes. In response to this need, the International Cannabis Consortium was established to combine the results of multiple GWASs to identify the genetic variants underlying individual differences in cannabis use phenotypes. Our rationale for focusing on lifetime cannabis use (yes/no) is because this phenotype is heritable and shares significant genetic risks with that risk for cannabis abuse or dependence.^14, 15, 33^ In contrast to frequency of use or abuse and dependence, which are not commonly assessed in large-scaled genetic studies, most general population studies have assessed lifetime cannabis use, thereby increasing our sample size and power to detect associations. Currently, the combined International Cannabis Consortium sample size for lifetime cannabis is 32 330 individuals from 13 cohorts from Europe, the United States and Australia, along with four independent replication samples comprising 5627 individuals. This sample size is considerably larger than the sample size of the previous GWAS investigating lifetime cannabis use in two samples from Australia and the UK, thereby providing substantially greater power to detect genetic variants of small effect size.

The aim of the present study is to identify genetic variants associated with lifetime cannabis use by meta-analysis of the GWAS results from all contributing International Cannabis Consortium samples. The tests of association for individual genetic variants will be complemented with gene-based tests of association. In addition, we will investigate which proportion of the heritability inferred by twin studies is explained by common SNPs captured on GWAS arrays. Finally, we will estimate the genetic correlation between lifetime cannabis and smoking initiation based on the analysis of our summary statistics and those from the publicly available Tobacco Alcohol and Genetics consortium.

## Materials and methods

### Cohorts

We performed a meta-analysis of GWA results from 13 discovery samples from Europe, USA and Australia including a total of 32 330 individuals of European ancestry. The size of the samples ranged from 721 to 6778 individuals. The age of the participants ranged from 16 to 87 years with an average of 34 years. The percentage of females ranged from 30 to 66% with an average of 53%. Owing to the differences in recruitment strategies, cultural and temporal difference, combined with likely variation in the drug availability between countries, there was a wide range in the prevalence of lifetime use (that is, never/ever used cannabis), which varied from 1 to 92% with an average of 44.5%.

Four additional independent samples with a total of 5627 subjects were used for replication. One sample (*n*=2660) consisted of African American subjects. The other three included subjects of European ancestry. See Table 1 for individual sample characteristics. The procedures for data collection per sample are described in the Supplementary Information 1.

### Phenotype and covariates

For all individuals, the data were available on whether or not the subject reported having ever used cannabis during their lifetime: yes (1) versus no (0). Although phrasing of the question slightly differed between samples (see Supplementary Information 1), our unit of analysis reflected lifetime cannabis use in all the samples.

Covariates included age at the time of phenotypic assessment, sex, birth cohort and principal components (obtained from the genome-wide genotype data). Spanning 20-year intervals, birth cohort was dummy coded, with the lowest birth cohort (that is, oldest age group) used as the reference group. The details about phenotypic assessment and individual sample characteristics for the discovery and replication samples are located in Supplementary Information 1 and Supplementary Table 1.

### Genotyping and imputation

Genotype imputation was based on the 1000 Genomes phase 1 reference panel.^34^ Allelic dosage data were used to account for genotype uncertainties. See Supplementary Table 2 for the genotyping platform, imputation program and quality control thresholds used.

### Statistical analyses

#### GWA analysis in each discovery cohort

The GWA analyses were performed by each group separately. Associations between the binary phenotype and the genotypes were tested genome-wide using a logistic regression model including covariates (see above). For family-based samples, familial relatedness was taken into account by using a sandwich correction as implemented in PLINK.^35^ The analyses plan can be found in Supplementary Information 3. It should be noted that some groups did do the analyses in a slightly different manner based on the characteristics of their sample. The analyses plan that was send to the participating groups is included in Supplementary Information 3. It should be noted that some groups did do the analyses in a slightly different manner based on the characteristics of their sample. Supplementary Table 2 lists the program used by each group.

#### Meta-analysis of GWAS results

Before performing the meta-analysis, we applied a set of filters to each GWA results set independently. First, we removed insertions and deletions, ensuring that all base pair positions were unique and referred to the same genetic variant (that is, SNP). Second, we removed genotyped SNPs that were not in Hardy–Weinberg equilibrium (*P*⩽10^−5^). Third, we removed SNPs with minor allele frequency (MAF) <√(5/N), which under the assumption of Hardy–Weinberg equilibrium corresponded to less than five estimated individuals in the least frequent genotype group. In the EGCUT1 sample, due to very low prevalence of lifetime cannabis use (1.3%), we excluded SNPs with MAF<0.2. Fourth, regardless of the quality score type used, we excluded SNPs with imputation quality scores below 0.6. Finally, SNPs present in only one sample and SNPs with alleles or allele frequencies inconsistent with the 1000 Genomes phase I European reference panel (absolute MAF difference >0.15) were removed.

We performed a fixed-effects meta-analysis based on the cohort's effect sizes and standard errors using METAL.^36^ Our meta-analysis combined association summary statistics for 6 444 471 SNPs that passed all the filters. We applied the conventional threshold of 5 × 10^−8^ as an indication of genome-wide significance (see ref. 37). Although the combined sample size of the meta-analysis based on the discovery samples is 32 330, the sample size per SNP varies due to missingness across subsamples.

#### Gene-based test

Results of the GWAS were then used as part of gene-based tests of association in the Knowledge-based mining system for Genome-wide Genetic studies (KGG) software package Version 3.5.^38, 39^ This approach uses an extended Simes test that integrates prior functional information and the meta-analysis association results when combining the SNP *P*-values within a gene to obtain an overall association *P*-value for each entire gene. We conducted 24 576 gene-based tests of association. The genome-wide significance level according to the Knowledge-based mining system for Genome-wide Genetic studies default setting of Benjamini and Hochberg false discovery rate threshold of 0.05 (ref. 40) was 9.38 × 10^−6^.

#### Estimation of SNP-based heritability and genetic overlap with lifetime cigarette smoking

The proportion of phenotypic variance that could be explained by the SNPs was estimated using the density estimation method developed by So *et al.*^41^ The density estimation method estimates the genome-wide distribution of effect sizes based on the difference between the observed distribution of test statistics in the meta-analysis and the corresponding null distribution. Before estimation, the SNPs present in at least 25% of the meta-analysis samples were pruned for LD. We used the *r*^2^=0.15 pruning level as the primary result for consistency with other applications of this method. Additional details are located in the Supplementary Information 2. LD Score regression^42, 43^ was used as an alternative method to estimate the SNP-based heritability, as well as to estimate the degree of genetic covariance between lifetime cannabis use (present study) and lifetime cigarette smoking^31^ (see Supplementary Information 2).

## Results

### Meta-analysis

No genome-wide significant associations between individual SNPs and lifetime cannabis use were observed (see Manhattan plot, Supplementary Figure 1a). However, the QQ plot (Supplementary Figure 1b) reveals strong enrichment of SNPs with *P*<10^−4^. Supplementary Figures 2a–m and 3a–m illustrate the Manhattan and QQ plots for each sample. Table 2 illustrates the top 10 independent (*R*^2^<0.1) SNPs associated with lifetime cannabis use. None of these 10 SNPs were replicated in the four independent replication samples (Supplementary Table 3). In a combined meta-analysis of the 10 top SNPs (that is, discovery plus replication samples), none of the SNPs reached genome-wide significance. Local plots of the most strongly associated regions, including neighboring genes, are provided in Supplementary Figures 4a–j. The most statistically significant marker (*P*-value =4.6 × 10^−7^) was rs4984460 located on chromosome 15 (see Supplementary Figure 5 for the forest plot). The SNP is located in an intergenic region between *LOC400456/LOC145820* and *NR2F2* and *MIR1469* genes. Supplementary Table 4 includes the 153 SNPs identified with *P*-values <10^−5^. Because not all SNPs passed the post-imputation quality control steps in all the samples, this table includes the effective sample size per SNP.

### Gene-based tests

**Figure 1:**
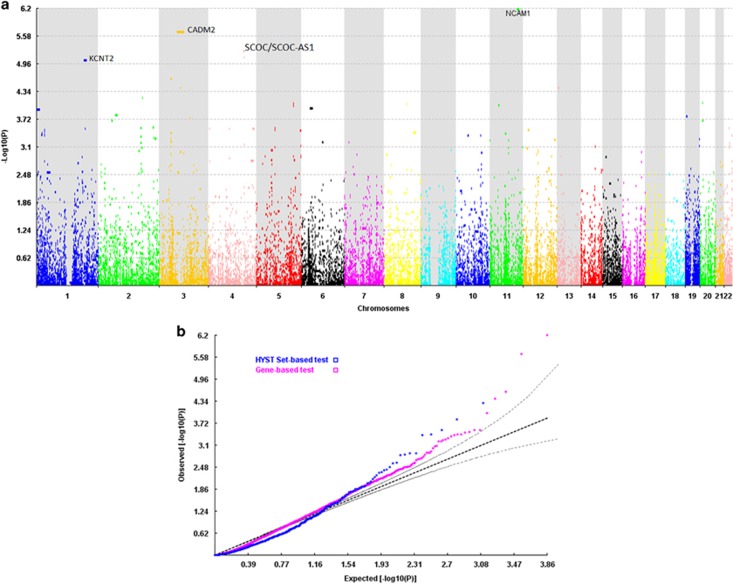
The Manhattan (**a**) and the QQ plot (**b**) based on results of the gene-based analysis performed in the discovery sample using HYST (hybrid set-based test).

The gene-based tests of associations were run on 24 576 genes/genetic regions (see ‘Materials and Methods' section for details). The Manhattan and QQ plot for this test are shown in Figures 1a and b. Results for the top 100 genes can be found in Supplementary Table 5. As shown in Table 3, four genes and one intergenic noncoding RNA region were significantly (false discovery rate-corrected *P*<0.05) associated with lifetime cannabis use: (i) neural cell adhesion molecule 1 (*NCAM1*, on 11q23); (ii) cell adhesion molecule 2 (*CADM2*, on 3p12); (iii) short coiled-coil protein (*SCOC*) and (iv) *SCOC* antisense RNA1 (*SCOC-AS1*, both located on 4q31); and (v) potassium channel, subfamily T, member 2 (*KCNT2*, on 1q31). Regional plots^44^ of these top genes are located in Supplementary Figure 6.

**Figure 2:**
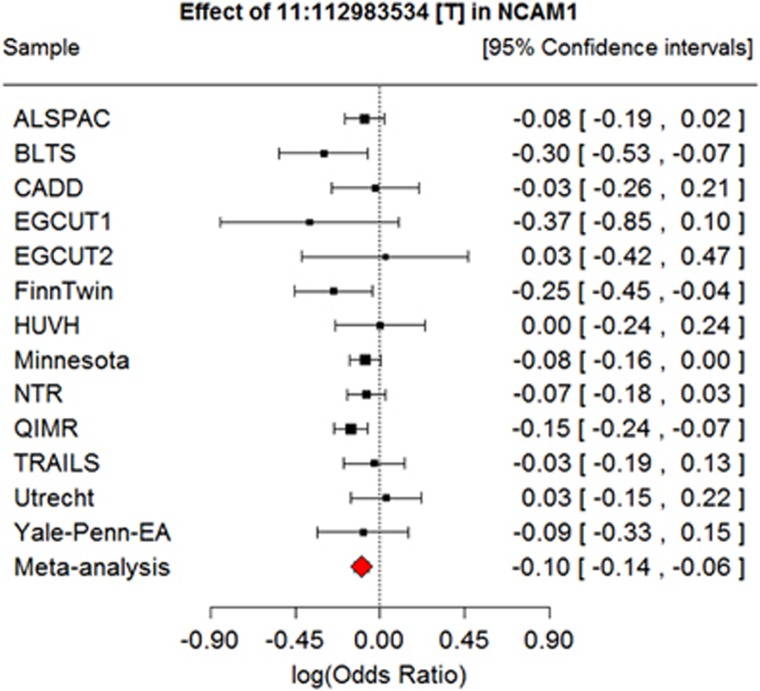
Forest plot for the top-SNP rs4471463 in the *NCAM1* gene on chromosome 11. SNP, single-nucleotide polymorphism.

The smallest gene-based *P*-value was found for the *NCAM1* gene. Within this gene, rs4471463 had the lowest SNP *P*-value, and was also among the top 10 associated SNPs. The forest plot in Figure 2 illustrates the effect of this SNP in each sample. In most samples, the effect is in the same direction, such that the major (T) allele is associated with a decreased risk of lifetime cannabis use. The forest plot for two SNPs with lowest *P*-values in the other significant gene regions can be found in Supplementary Figure 5.

Of the five genes included in our replication analyses, none were replicated in two of the independent replication samples (see Table 3). In the African American replication sample, suggestive associations with *SCOC-AS1* (*P*=0.044) and *SCOC* (*P*=0.027) were found.

### SNP-based heritability and genetic overlap with lifetime cigarette smoking

Using the density estimation method (see ‘Materials and Methods' section for a description), all the SNPs available in at least 25% of the samples when combined explained 20% of the total variance in lifetime cannabis use (*P*<0.001). Alternative estimation with LD score regression also yielded a significant heritable component of 13% (*h*^2^_LD_=0.13, s.e.=0.02, *P*=1.4 × 10^−7^). These variance estimates were robust across pruned sets with similar *r*^2^ thresholds (see Supplementary Table 6). Stricter LD pruning (that is, *r*^2^=0.05), or restricting analyses to SNPs present in all studies substantially decreased the estimate of variance explained. Both SNP heritability estimates confirmed that lifetime cannabis use has a significant heritable component (13–20%), indicating that GWAS should be able to identify these common SNPs (but effect sizes are small and large sample sizes are thus required). However, because these estimates are only based on common SNPs, the total heritability of lifetime cannabis use is likely to be higher.

The LD score regression analyses revealed a strong and highly significant genetic correlation (*r*_g_=0.83, s.e.=0.15, *P*=1.85 × 10^−8^) between lifetime cannabis use and lifetime cigarette smoking (based on the Tobacco Alcohol and Genetics consortium^31^ summary results), implying that SNPs for lifetime cannabis use and lifetime cigarette smoking are highly correlated.

## Discussion

To date, this is the largest GWA study of lifetime cannabis use. We performed meta-analysis of the GWA results based on a discovery sample comprising 32 330 individuals from 13 cohorts, and a replication sample comprising 5627 subjects from four cohorts (including one African American cohort). There were no genome-wide significant SNP associations. However, heritability analyses revealed that between 13 and 20% of the variation in lifetime cannabis use could be explained by common SNPs. Moreover, gene-based tests of association identified four protein-coding genes and one intergenic region significantly associated with lifetime cannabis use including *NCAM1,* which has previously been linked to substance use.^45, 46, 47, 48^ Finally, we revealed that the genetic liability to lifetime cannabis use correlated to a large extent (*r*=0.83) with the genetic liability to lifetime cigarette smoking. Our results are consistent with the hypothesis that lifetime cannabis use is a highly polygenic trait, comprising many SNPs each with small effects contributing to lifetime risk. Moreover, portions of the genetic risk in lifetime cannabis use likely correlates with other substances including cigarette smoking.

Our top gene associated with lifetime cannabis use was *NCAM1*, a known candidate for nicotine dependence.^45^ The role of *NCAM1* is to regulate pituitary growth hormone secretion as a membrane-bound glycoprotein that mediates cell–cell contact by hemophilic interactions.^46^ *NCAM1* is part of the *NCAM1*–*TTC12*–*ANKK1*–*DRD2* (NTAD) gene cluster, which is related to neurogenesis and dopaminergic neurotransmission. Importantly, the NTAD cluster has been associated with smoking behavior and nicotine dependence,^45, 47, 48, 49, 50, 51, 52^ alcohol dependence,^53, 54^ heroin dependence,^55^ as well as other substance use disorders.^54^ Although it is plausible that *NCAM1* is capturing pleiotropic risks underpinning the liability to licit and illicit substance use in general, we note that *NCAM1* was not identified either by the Tobacco Alcohol and Genetics consortium or other consortia for cigarette smoking.^30, 31, 32^ The functions of the putative variants responsible for the associations in the candidate-gene studies remain to be determined.

The second gene, *CADM2*, is a synaptic cell adhesion molecule (SynCAM family) belonging to the immunoglobulin (Ig) superfamily. Variants in the *CADM2* gene have been previously associated with body mass index,^56^ processing speed^57^ and autism disorders.^58^ Interestingly, these phenotypes were associated with cannabis use in previous studies,^59, 60, 61^ which together suggest that *CADM2* can be considered an important gene related to a variety of complex traits. It is possible that the association with lifetime cannabis use may be driven, for example, by differences in personality rather than as a direct relationship with lifetime use.

The third gene, *SCOC,* encodes a short coiled-coil domain-containing protein that localizes to the Golgi apparatus. Many coiled-coil-type proteins are involved in important biological functions such as the regulation of gene expression through the regulation of transcription factor binding.^62^ The function of *SCOC* is largely unknown and no previous association studies have linked *SCOC* to cannabis or other substance use phenotypes. The *SCOC* antisense RNA1 gene is located in the same chromosomal region.

Finally, *KCNT2* encodes a potassium voltage-gated channel (subfamily S, member 2). The sodium-activated potassium channels Slack and Slick are encoded by *KCNT1* (potassium channel, subfamily T, member 1) and *KCNT2*, respectively, which are found in neurons throughout the brain. Suggestive association for SNPs near KCNT2 have previously been found for cocaine dependence and for early-onset, highly comorbid, heavy opioid use.^63, 64^ This suggests that potassium signaling may have a role in addiction.

The lack of genome-wide significant associations for individual SNPs is consistent with previous GWA studies of lifetime cannabis use^26, 27^ and cannabis dependence.^25^ The difficulty of identifying specific SNPs for lifetime cannabis use may be attributable to several reasons. First, complex traits are known to be influenced by many variants, each with very small effect sizes. Although power calculations reveals suitable power (96%) to detect odds ratios of 1.15 based on common SNPs (MAF=0.2), the power to detect smaller effect sizes remains lower. For example, there is only 28% power to detect effect sizes with odds ratio of 1.1 and MAF=0.2. Therefore, our data suggest that the effect sizes of single variants contributing to lifetime cannabis use are likely to be smaller than 1.15. Combining variants within larger units (that is, genes) did however reveal four significant genes associated with lifetime cannabis use implying that these genes are appropriate targets for future functional studies of cannabis use. Unfortunately, our gene-based results were not replicated in the replication samples, probably due to low sample sizes and therefore low power. In the African American replication sample, we did find suggestive association with *SCOC-AS1* and *SCOC*.

On the basis of twin studies, the heritability of lifetime cannabis use is estimated at 40–50%.^12^ In our study, all common SNPs combined explained 13–20% of the variance in the liability to use cannabis depending on the method used. Stricter LD pruning (that is, *r*^2^=0.05) or restricting to SNPs observed (genotyped or imputed) in all the analyses, substantially reduces the estimate of variance explained. Speculatively, this may indicate that much of the variance explained comes from SNPs located in the regions of weak LD. Such effects are likely to be poorly tagged for the estimation of variance explained after strict LD pruning, and are likely to be more difficult to impute owing to a lack of strongly correlated genotyped SNPs (and thus missing from some studies). Our SNP-based heritability estimates lie in between two previous heritability estimates for lifetime cannabis use based on the Genome-wide Complex Trait Analysis^65^ software package. Verweij *et al.*^26^ estimated that 6% of the variance in lifetime cannabis use is explained by aggregated common SNPs (MAF>0.05). Minică *et al.*^27^ found an estimate of 25%. Provided that the current sample is much larger than the samples used in the previous studies, we conclude that approximately one-third to half of the heritability is explained by common SNPs captured on a GWAS array. Other sources of variation may explain the discrepancy between SNP- and twin-based heritability estimates. For example, age-related genetic differences, non-additive genetic variance, interactions between genetic variants and environmental risk factors, epistasis and/or rare mutations may also have a role.

Our results indicate a very high genetic overlap (*r*=0.83) between our measure of lifetime cannabis use and lifetime cigarette use when based on the SNP panel. Twin studies have shown moderate to high genetic correlations of 0.59–0.74 between lifetime cannabis and nicotine use.^66^ Kendler *et al.*^67^ also reported significant biometrical genetic correlations between the levels of cannabis, nicotine and alcohol use, which were increasingly influenced by common genetic risks detectable in early adulthood.

Our findings should be interpreted in the context of at least four potential limitations. First, our study was underpowered to detect very small effects of individual variants. Power analyses revealed that a twofold increase in sample size is required to detect SNP effect sizes with odds ratios of 1.1. Second, lifetime cannabis use is a dichotomous measure combining single lifetime, regular and chronic users. Consequently, our sample may compromise heterogeneous patterns of use, which has the potential to reduce the power to detect genetic association.^68^ Third, prevalences of lifetime cannabis use varied between 1% (EGCUT1) and 92% (Yale Penn EA). This was likely due to differences in the sample characteristics, recruitment strategies and the political differences between countries. Despite these differences, the forest plots of the key SNPs (see Figure 2; see also Supplementary Figure 5) revealed that the 95% confidence intervals surrounding the effect estimates typically included the estimated meta-analytic effect, which tends to overlap across studies. This indicates that the input samples were representative of the same population of users. Finally, the average age of participants varied between 18 (ALSPAC) and 45 (QIMR) years. Consequently, some younger participants might have initiated cannabis use at a later age, but have been classified as ‘never users' in the current study. This can decrease power, but does not invalidate our results. In addition, we note that the average age of each sample did not correlate with sample prevalences (*r*=−0.04, *P*=0.91).

On the basis of our observations, the following recommendations for future studies can be made. We have identified four genes significantly associated with cannabis use, which are candidates for follow-up functional studies. In particular, the role of *NCAM1* can be examined to determine the functional role of this gene, possibly in combination with other genes in the same gene cluster (*NCAM1*–*TTC12*–*ANKK1*–*DRD2*).

The next goal of the International Cannabis Consortium is to perform a meta-analysis on GWA studies investigating the age at first cannabis use. Our rationale is based on the observation that early initiation of cannabis use is associated with rapid progression towards cannabis abuse and dependence, polysubstance use and other substance use disorders.^69, 70, 71^ Methods other than GWASs may also be used to reveal the biological pathways of cannabis use, such as rare variant association analyses. The environmental risk factors may be incorporated to investigate gene × environment interactions. Hopefully, the combination of advanced technologies and novel statistical approaches with larger samples will further contribute to our understanding of the genetic architecture of cannabis use.

## Conclusion

We have performed the largest meta-analysis to date of GWASs investigating cannabis use phenotypes. With a sample of over 32 000 individuals, our results implicate four genes as involved in lifetime cannabis use: *NCAM1*, *CADM2*, *SCOC* and *KCNT2*. Our results illustrated that lifetime cannabis use is under the influence of many common genetic variants. The combined SNPs explained 13–20% of the phenotypic variation, and revealed a high degree of genetic sharing (*r*=0.83) with lifetime cigarette smoking. Future studies should investigate the impact of these genes on the biological mechanisms leading to lifetime cannabis use.

## Figures and Tables

**Table 1:** Discovery and replication sample characteristics

*Sample*	*Country*	N	*% Users*	*% Female*	*Mean age (range)*	N *SNPs*
*Discovery*						
ALSPAC	UK	2976	42	56	18 (17–19)	5 182 231
BLTS	Australia	721	60	57	26 (18–33)	4 558 509
CADD	USA	853	79	30	25 (18–36)	4 972 726
EGCUT1	Estonia	2765	1.3	55	34 (18–66)	6 048 479
EGCUT2	Estonia	970	4.8	51	31 (18–50)	5 171 164
FinnTwin	Finland	1029	27	52	23 (20–29)	4 364 135
HUVH	Spain	981	20	30	36 (17–87)	4 971 170
MCTFR	USA	6241	59	54	37 (18–71)	6 304 767
NTR	Netherlands	4653	27	66	37 (18–60)	4 644 238
QIMR	Australia	6778	51	54	45 (18–85)	5 901 727
TRAILS	Netherlands	1226	51	47	19 (18–21)	5 336 901
Utrecht	Netherlands	1173	54	54	21 (18–37)	4 831 885
Yale Penn EA	USA	1964	92	40	38 (16–76)	5 856 902
						
*Replication*						
Radar	Dutch	338	59	44	20 (17–22)	10
SYS	Canada	551	51	56	50 (36–65)	10
TwinsUK	UK	2078	12	93	58 (18–86)	10
Yale Penn AA	US	2660	82	46	42 (16–76)	10

Abbreviations: *N*, sample size; N SNPs, number of SNPs used for the meta-analysis; SNP, single-nucleotide polymorphism; % female, percentage of females; % users, percentage of users that ever used cannabis.

**Table 2:** Top 10 SNPs with meta-analysis results of discovery samples, and results of combined discovery and replication samples

*SNP*	*Chr*	*BP (hg19)*	*A1*	*A2*	*Freq A1*	*Discovery*			*Combined*^a^	
						*Beta (s.e.)*	P-*value*	*Direction*^b^	*Beta (s.e.)*	P-*value*
rs4984460	15	96424399	T	G	0.75	−0.11 (.023)	4.6 × 10^−^^7^	+−−++−−−−−−−+	−0.11 (0.023)	2.2 × 10^−6^
rs2099149	12	30479358	T	G	0.81	−0.16 (0.032)	9.8 × 10^−7^	−−−?−??−?−−+−	−0.17 (0.034)	5.1 × 10^−7^
rs7675351	4	141218757	A	C	0.86	−0.15 (0.031)	1.4 × 10^−6^	−−−?+−−−?−−−−	−0.13 (0.033)	1.1 × 10^−4^
rs4471463	11	112983595	T	C	0.55	−0.09 (0.020)	1.5 × 10^−6^	−−−−+−+−−−−+−	−0.1 (0.021)	9.0 × 10^−7^
rs7107977	11	915764	A	G	0.60	0.27 (0.058)	1.9 × 10^−6^	??+++?+???+?+	0.29 (0.064)	6.4 × 10^−6^
rs58691539	2	52753909	T	G	0.91	−0.29 (0.062)	2.1 × 10^−6^	−????−?−????−	−0.29 (0.062)	2.2 × 10^−6^
rs2033867	2	175188281	A	G	0.06	0.24 (0.051)	2.6 × 10^−6^	+??????+++??+	0.23 (0.050)	4.2 × 10^−6^
rs35053471	3	47124761	A	T	0.38	−0.10 (0.022)	2.7 × 10^−6^	−−−−−?+−−−−−−	−0.09 (0.022)	9.2 × 10^−5^
rs12518098	5	60864467	C	G	0.68	0.10 (0.022)	3.0 × 10^−6^	++++−++++++++	0.09 (0.023)	4.7 × 10^−5^
rs73067624	1	196333461	T	C	0.90	−0.18 (0.039)	3.1 × 10^−6^	−?−?−−−−?−−−−	−0.16 (0.041)	6.3 × 10^−5^

Abbreviations: A1, allele 1; A2, allele 2; BP (hg19), location in base pairs in human genome version 19; Chr, chromosome; Freq A1, frequency of allele 1; SNP, single-nucleotide polymorphism.

a The combined sample contains the discovery samples and the Radar, SYS and TwinsUK replication samples.

b Direction per sample: allele A1 increases (+) or decreases (−) liability for cannabis use, or sample did not contribute to this SNP because it did not pass the post-imputation quality control (?). Order of samples: ALSPAC, BLTS, CADD, EGCUT1, EGCUT2, FinnTwin, HUVH, MCTFR, NTR, QIMR, TRAILS, Utrecht, Yale Penn EA. Sample information can be found in Table 1.

SNPs are displayed when not in linkage disequilibrium (*R*^2^<0.1. For SNPs with *R*^2^⩾0.1, only the most significant SNP is shown in the top 10).

**Table 3:** Top five genes from the gene-based tests of association with corrected *P*-values (Benjamini and Hochberg) based on the meta-analytic discovery and replication samples

*Gene*	*Chr*	*Start position (hg19)*	*BP length*	N *SNPS*	*Nominal* P-*values discovery*	*Corrected* P-*values discovery*	*Nominal* P-*values EU replication samples*	*Nominal* P-*values replication African Americans*
*NCAM1*	11	112831968	303 952	400	6.26 × 10^−7^	0.015	0.381	0.302
*CADM2*	3	85008132	1 115 448	978	2.13 × 10^−6^	0.026	0.744	0.112
*SCOC-AS1*	4	141204879	89 668	81	5.76 × 10^−6^	0.046	0.681	0.044
*SCOC*	4	141264614	39 097	111	7.85 × 10^−6^	0.046	0.636	0.027
KCNT2	1	196194909	382 653	237	9.38 × 10^−6^	0.046	0.269	0.201

Abbreviations: BP length, base pair length; chr, chromosome; hg19, human genome version 19; *N* SNPs, number of SNPs used for the meta-analysis; SNP, single-nucleotide polymorphism.

## Notes

HRK is a consultant or Advisory Board Member for Alkermes, Lilly, Lundbeck, Otsuka, Pfizer, Roche; member of the American Society of Clinical Psychopharmacology's Alcohol Clinical Trials Initiative, supported by AbbVie, Ethypharm, Lilly, Lundbeck and Pfizer. The remaining authors declare no conflict of interest.
