# Supplementary material for: Genome-wide association study of lifetime cannabis use based on a large meta-analytic sample of 32 330 subjects from the International Cannabis Consortium
Source: Transl Psychiatry. 2016 Mar 29;6(3):e769–. doi: 10.1038/tp.2016.36 (PMC4872459; doi:10.1038/tp.2016.36)
Supplement: Supplementary Information 1 [file tp201636x1.doc]

# Supplemental Information 1. Information about sample collection

# Discovery samples

## ALSPAC: Avon Longitudinal Study of Parents and Children — United Kingdom

The Avon Longitudinal Study of Parents and Children (ALSPAC) is a prospective cohort study which recruited 14,541 pregnant women residing in Avon, United Kingdom, with expected dates of delivery between 1 April 1991 and 31 December 1992. Of these initial pregnancies, there was a total of 14,676 fetuses, resulting in 14,062 live births and 13,988 children who were alive at 1 year of age. Full details of study recruitment and methodology have been published previously^1^. Detailed information on the mothers and their children has been collected from self-report questionnaires and attendance at clinics. Please note that the study website contains details of all the data that is available through a fully searchable data dictionary (http://www.bris.ac.uk/alspac/researchers/data-access/data-dictionary/).Ethical approval for the study was obtained from the ALSPAC Ethics and Law Committee and the Local Research Ethics Committees. Data on cannabis use for this project was measured in the ALSPAC offspring between 17 and 19 years of age and limited to unrelated individuals only. The answer categories were recoded as ever (1) versus never (0) use of cannabis. Individuals were also asked about frequency of cannabis use if they answered yes to ever having used cannabis. Cannabis data were available for 4,303 individuals. Both genotype and lifetime cannabis use data were available for 2,976 individuals.

References:

1. Boyd et al. (2013), Cohort profile: The ‘Children of the 90s’ - the index offspring of the Avon Longitudinal Study of Parents and Children. *IJE, 42*, 111-127.

## BLTS: Brisbane Longitudinal Twin Study - Australia

Beginning in 1992, the Brisbane Longitudinal Twin Study (BLTS) consists of 3,561 individuals: 1,422 twin pairs and 717 additional siblings first enrolled at age 12 years and now aged 30 years and older (Gillespie, 2013). The sample is: genetically informative (MZ and DZ twins, and often parents and siblings; genotyped for 610,000 common single nucleotide polymorphisms - SNPs); (b) large; (c) longitudinal with many participants have been assessed at 12, 14, 16 and 21 years of age; (d) well characterized for behavioral and brain-related outcomes; (e) rich in biological samples; and includes (f) a subgroup [n=969] who have undergone MRI scanning. As part of an ongoing US NIH/NIDA funded project beginning 2009, measures of lifetime cannabis use, abuse and dependence data are collected, along with diagnostic data for nicotine, alcohol, and other illicit substances, as well as pilot epidemiological data for ecstasy and methamphetamine use. The average age at interview is 25.65 yrs (SD=3.65, range=18-38yrs). Lifetime cannabis use was assessed by asking twins, “In your life, have you ever used cannabis (marijuana, pot, grass or hash)?” The entire BLTS sample and 1,549 of their parents have GWAS data (Illumina 610k chip) (Medland et al., 2009) imputed on the GRCh37 assembly. The final sample included 721 individuals with both genotypic and life cannabis use data.

References

Gillespie, N.A., A.K. Henders, T.A. Davenport, D.F. Hermens, M.J. Wright, N.G. Martin, and I.B. Hickie, The Brisbane Longitudinal Twin Study: Pathways to Cannabis Use, Abuse, and Dependence project-current status, preliminary results, and future directions. Twin Res Hum Genet, 2013. 16(1): p. 21-33. PMC3805122

Medland SE, Nyholt DR, Painter JN, McEvoy BP, McRae AF, Zhu G et al. Common variants in the trichohyalin gene are associated with straight hair in Europeans. Am J Hum Genet 2009; 85(5): 750-755.

## CADD: Center on Antisocial Drug Dependence - United States

Data on cannabis use were collected as part of various studies contained within the Center on Antisocial Drug Dependence (CADD). Genotypes were available from 1901 unrelated individuals who were over-selected for adolescent behavioral disinhibition; of these, 1031 were of solely Caucasian ancestry. Among genotyped Caucasian participants, lifetime cannabis use had been collected from 853 individuals who were 18 years of age or older as of May 2013. Lifetime cannabis use data were collected using a supplemental questionnaire appended to the CIDI-SAM, which asked participants to self report "Have you ever used ________ ?" (yes/no) for each of 14 substances (plus "other"), including marijuana. If participants answered in the affirmative, they were asked to respond to questions about age at first use, age at regular use, typical pattern of use, and days used within the past six months; otherwise, participants were instructed to skip to questions about the next substance. If participants had been assessed more than once, lifetime cannabis use data from their most recent (i.e. oldest age) assessment was used for the current analysis.

**Reference**

Derringer, J., Corley, R. P., Haberstick, B. C., Young, S. E., Demmitt, B., Howrigan, D. P., Kirkpatrick, R. M., Iacono, W. G., McGue, M., Keller, M., Brown, S., Tapert, S., Hopfer, C. J., Stallings, M. C., Crowley, T. J., Rhee, S. H., Krauter, K., Hewitt, J. K., McQueen, M. B. (under review). Genome-wide association study of behavioral disinhibition in a selected adolescent sample.

## EGCUT1&2: Estonian Genome Center University of Tartu - Estonia

The Estonian cohort is from the population-based biobank of the Estonian Genome Project of University of Tartu (EGCUT). The project is conducted according to the Estonian Gene Research Act, and all participants have signed the broad informed consent. The current cohort size is >51,515, 18 years of age and older, which reflects closely the age distribution in the adult Estonian population. Subjects are recruited by the general practitioners (GP) and physicians in the hospitals were randomly selected from individuals visiting GP offices or hospitals. Each participant filled out a computer-assisted personal interview during 1-2 hours at a doctor’s office, including personal data (place of birth, place(s) of living, nationality. etc.), genealogical data (three generation family history), educational and occupational history, and lifestyle data (physical activity, dietary habits, smoking, alcohol consumption, women’s health, quality of life). Data on lifetime cannabis use was collected during the interview. The question asked was, if the person has used other substances besides tobacco and alcohol and it was possible to add a note to describe the exact substances and if the person has used them once or many times. Cannabis user cases were selected as people, who had used cannabis for more than once.

## FinnTwin: Finnish Twin Cohort (FinnTwin12 & FinnTwin16) - Finland

**In FinnTwin12 (FT12),** data on lifetime cannabis use were collected as part of a longitudinal study targeting all Finnish twin pairs born in 1983-1987 (Kaprio, Pulkkinen, Rose 2002). Four waves of data collection have been completed (at ages 12, 14, 17.5, and in early adulthood age range 21-25) (Kaprio, 2013). In wave 4, using a SSAGA interview we asked whether participants ever experimented with cannabis (no, yes) and if yes, at what age they first experimented with cannabis. They were also asked how many times they have used cannabis. Cannabis abuse and dependence were also assessed. Lifetime cannabis use data were available for 1346 FT12 subjects (25.3% (N=341) ever users). Both genotype and cannabis data were available for 929 FT12 subjects (26.5% (N=247) ever users).

**In FinnTwin16 (FT16)**, data on lifetime cannabis use were collected as part of a longitudinal study targeting all Finnish twin pairs born in 1975-1979 (Kaprio, Pulkkinen, Rose, 2002). Five waves of data collection have been completed (at ages 16, 17, 18.5, mean age 24, and mean age 34). In wave 4 we conducted SSAGA interviews from a subsample. In the SSAGA interview we asked whether participants ever experimented with cannabis (no, yes) and if yes, at what age they first experimented with cannabis. They were also asked how many times they have used cannabis. Cannabis abuse and dependence were also assessed. Lifetime cannabis use data were available for 602 FT16 subjects (34.2% (N=206) ever users). Both genotype and cannabis data were available for 100 FT16 subjects (36.0% (N=36) ever users).

Altogether both genotype and cannabis data were available for 1029 subjects (27.5% (N=283) ever users).

**References**

Kaprio J. The Finnish Twin Cohort Study: an update. Twin Res Hum Genet. 2013;16(1):157-62.

Kaprio J, Pulkkinen L, Rose RJ. Genetic and environmental factors in health-related behaviors: studies on Finnish twins and twin families. Twin Res. 2002;5(5):366-71.

## HUVH: Hospital Universitari Vall d'Hebron – Barcelona - Spain

Data on lifetime cannabis use were collected as part of a GWAS study of persistent ADHD. Recruitment of participants was performed between 2004 and 2011 at the Department of Psychiatry of the Hospital Universitari Vall d’Hebron, Barcelona, Spain. Genotypes were available from 1039 unrelated Caucasian individuals. Lifetime cannabis use data were collected from 981 of them (20% cannabis users) and assessed by the Structured Clinical Interview for DSM-IV Axis I and Axis II Disorders (SCID-I and SCID-II). The average age at assessment was 35.6 years (SD = 11.3), 70% of participants were males and 61% met Diagnostic and Statistical Manual of Mental Disorders, 4^th^ Edition (DSM-IV) criteria for ADHD. The study was approved by the ethics committee of the institution and informed consent was obtained from all subjects.

**Reference**

Sánchez-Mora C, Ramos-Quiroga JA, Bosch R, Corrales M, Garcia-Martínez I, Nogueira M, Pagerols M, Palomar G, Richarte V, Vidal R, Arias-Vasquez A, Bustamante M, Forns J, Gross-Lesch S, Guxens M, Hinney A, Hoogman M, Jacob C, Jacobsen KK, Kan CC, Kiemeney L, Kittel-Schneider S, Klein M, Onnink M, Rivero O, Zayats T, Buitelaar J, Faraone SV, Franke B, Haavik J, Johansson S, Lesch KP, Reif A, Sunyer J, Bayés M, Casas M, Cormand B, Ribasés M. Case-control genome-wide association study of persistent attention-deficit hyperactivity disorder identifies FBXO33 as a novel susceptibility gene for the disorder. Neuropsychopharmacology. 2015 Mar;40(4):915-26. doi: 10.1038/npp.2014.267.

Ramos-Quiroga JA, Sánchez-Mora C, Casas M, Garcia-Martínez I, Bosch R, Nogueira M, Corrales M, Palomar G, Vidal R, Coll-Tané M, Bayés M, Cormand B, Ribasés M. Genome-wide copy number variation analysis in adult attention-deficit and hyperactivity disorder. J Psychiatr Res. 2014 Feb;49:60-7.

MCTFR: Minnesota Center for Twin and Family Research – United States

Data on lifetime cannabis use were collected during the course of several longitudinal family studies. Youth offspring and their parents participated in phone interviews or laboratory visits to the MCTFR facilities in Minneapolis, Minnesota. The MCTFR focuses on two major projects: Minnesota Twin Family Study (MTFS) and Sibling Interaction and Behavior Study (SIBS). The MTFS sample consists of like-sex twin offspring and their parents. Recruitment of participants occurred between 1990 and 2006. Twins were aged 11 or 17 at their intake assessment, and have been followed up regularly into their third decade (generally up to age 29). The SIBS sample contains dyads of non-twin adolescent siblings. Youth and their caregivers were recruited between 1998 and 2004. Siblings were followed up a maximum of two times and, at last contact, ranged between ages 19 and 26. Parents in both samples tended to be middle-aged at their intake assessment (46.3 ± 5.4 years).

The starting sample in these MCTFR studies was 9,859 individuals. Several different instruments, including semi-structured interviews and computerized self-report surveys, were administered to assess cannabis use. Participants were asked if they had ever used cannabis and, in the event of an affirmative response, were further questioned about the frequency and duration of use. Lifetime cannabis use data were available for 9,817 participants (of whom 9,110 were over the age of 18). Both genotype and cannabis data were available for 6,241 Caucasian adults.

## NTR: Netherlands Twin Register — The Netherlands.

Data on lifetime cannabis use were collected as part of a longitudinal study on health, personality and lifestyle in adolescent and adult twins and their relatives (i.e., their non-twin siblings, parents, spouses and children). Nine waves of data collection have been completed (in 1991, 1993, 1995, 1997, 2000, 2002, 2004 and 2009-2010, 2011-2012) and the 10^th^ wave is ongoing (2013-present). Data on cannabis use were administered 4 times in 1995 (wave 3), 1997 (wave 4), 2000 (wave 5) and 2011-2012 (wave 8). Questions on cannabis are also asked in the 10^th^ wave, but data are not yet available. In wave 3, 4 and 5 the participants were asked at what age they experimented with cannabis for the first time and at what age they regularly used cannabis. The answer categories were: 1=never, 2=11 years or younger, 3=12 years, 4=13 years, 5-14 years, 6=15 years, 7=16 years, 8=17 years, 9=18 years or older in wave 2 and 3, and 1=11 years or younger, 2=12-13 years, 3=14-15 years, 4=16-17 years, 5=18 years or older and never. The answer categories were recoded to ever (1) versus never (0) used cannabis. In wave 8 we asked whether participants ever experimented with cannabis (no, yes) and if yes, at what age they started. They were also asked whether they ever used cannabis on a regular basis (no, yes) and if yes, at what age. If subjects participated more than once, the answers were checked for consistency. Both genotype and lifetime cannabis use data were available for 4653 subjects.

## QIMR: Queensland Institute of Medical Research Berghofer adults — Australia

Data from Australian adults were collected in twin family studies conducted at the QIMR Berghofer Medical Research Institute. Data on cannabis use were obtained from: 1) a series of studies conducted collaboratively by Nick Martin and Andrew Heath between 2001 and 2006 (Pergadia et al., 2009; Saccone et al., 2007; Distel et al., 2008), and 2) a study conducted between 1996 and 2000 of 6233 twin individuals from the young adult cohort (born between 1964 and 1971) (see Nelson et al. 2002; Knopik et al. 2004). In both studies individuals participated in semi-structured telephone interviews primarily focussed at psychiatric disorders. The interview was an adaptation of the SSAGA (Semi-Structured Assessment for the Genetics of Alcoholism). As part of this interview individuals were asked whether they had ever used cannabis. In case individuals participated in both studies, data from the last assessment were included.

The genotypic data are derived from multiple waves of genotyping. DNA samples were collected in accordance with standard protocols and submitted to different genotype centres using different Illumina SNP platforms (317 single, 370 single, 370 duo, 670 quad, 610 quad) (see Medland et al., 2009). Phenotypic and genotypic data collections were approved by the QIMR Human Research Ethics Committee and informed consent was obtained from all participants.

The final sample included 6778 individuals with both genotype and phenotype data (NB. One twin per MZ twin pair was deleted).

**References**

Distel MA, Trull TJ, Derom CA, Thiery EW, Grimmer MA, Martin NG *et al.* Heritability of borderline personality disorder features is similar across three countries. *Psychol Med* 2008; 38(9): 1219-1229.

Knopik VS, Heath AC, Madden PAF, Bucholz KK, Slutske WS, Nelson EC, Statham D, Whitfield JB, Martin NG (2004). Genetic effects on alcohol dependence risk : re-evaluating the importance of psychiatric and other heritable risk factors. Psychological Medicine 34, 1519–1530.

Medland SE, Nyholt DR, Painter JN, McEvoy BP, McRae AF, Zhu G *et al.* Common variants in the trichohyalin gene are associated with straight hair in Europeans. *Am J Hum Genet* 2009; 85(5): 750-755.

Nelson EC, Heath AC, Madden PAF, Cooper ML, Dinwiddie SH, Bucholz KK, Glowinski A, McLaughlin T, Dunne MP, Statham DJ, Martin NG (2002). Association between self-reported childhood sexual abuse and adverse psychosocial outcomes: results from a twin study. Archives of General Psychiatry 59, 139–145.

Pergadia ML, Agrawal A, Loukola A, Montgomery GW, Broms U, Saccone SF. Genetic linkage findings for DSM-IV nicotine withdrawal in two populations. *Am J Med Genet B* 2009; 150B: 950-959.

Saccone SF, Pergadia ML, Loukola A, Broms U, Montgomery GW, Wang JC *et al.* Genetic linkage to chromosome 22q12 for a heavy-smoking quantitative trait in two independent samples. *Am J Hum Genet* 2007; 80(5): 856-866.

## TRAILS: TRacking Adolescents’Individual Lives Survey — The Netherlands.

Data on lifetime cannabis use were collected as part of a Dutch longitudinal study on the development of (mental) health in adolescence and young adulthood. Four waves of data collection have been completed to date; data on lifetime cannabis use were collected at the fourth wave, in 2008-2010, when the sample was 18-20 years old. The participants were asked if and how often they had used cannabis during (1) their lives, (2) the last year, and (3) the last month; and how old they were when they used cannabis for the first time. The answer categories were recoded to ever (1) versus never (0) used cannabis. Lifetime cannabis use data were available for 1696 subjects. Both genotype and cannabis data were available for 1226 subjects.

## Utrecht: Utrecht Cannabis Cohort (CannabisQuest) –The Netherlands

Participants were recruited using a project website launched in 2006 targeted at Dutch young adults and adolescents from 18 to 25 years (www.cannabisquest.nl) (Schubart et al., 2010). Strategies to generate traffic on the project website included collaboration with over a hundred colleges, universities, and youth centres, as well as the use of online commercial advertisement products (i.e. banners and text links) (Schubart et al., 2010). The chance to win an Apple iPod™ or a Nintendo Wii™ was used as an incentive. Double entries were prevented by exclusion of subjects with an identical e-mail address, surname, and date of birth. Anonymous submission of data was not possible. The online assessment included verification questions to protect against random answers, and participants failing to correctly complete the verification questions were subsequently excluded. From the online data (N = 17,698), 1259 participants were included for subsequent genetic assessment in two waves. First, in order to increase power for gene × environment interactions (Boks et al., 2007), we prioritized a sample of 719 participants who belonged to the top or bottom quintile of total scores of psychotic experiences as measured by the Community Assessment of Psychic Experiences (CAPE) score (see below) that were either cannabis naïve (i.e. a lifetime cannabis exposure frequency less than 6 times) or were heavy cannabis users (i.e. current expenditure for personal cannabis use exceeded 3€ weekly). Second, an unselected sample of 540 individuals was included. As ascertained with the validated Dutch version of either the Structured Clinical Interview (SCID) (First et al., 1997) or the MINI International Neuropsychiatric Interview (Sheehan et al., 1998), healthy controls had no history of any psychotic disorder. The possible concomitant use of recreational drugs was assessed with the substance abuse module of the Composite International Diagnostic Interview (Compton, 1993). Participants provided a urine sample to screen for the presence of recreational drugs in order to verify recent self-reported cannabis use. The study was approved by the Ethical Review Board of the University Medical Center Utrecht and all participants gave written informed consent. For a total of 1173 participants data on lifetime cannabis use and genotypes were available.

**References**

Schubart CD, van Gastel WA, Breetvelt EJ, Beetz SL, Ophoff RA, Sommer IE, Kahn RS, Boks MP. Cannabis use at a young age is associated with psychotic experiences. Psychol Med 2011;41:p 1301-1310.

Vinkers CH, Van Gastel WA, Schubart CD, Van Eijk KR, Luykx JJ, Van Winkel R, ; GROUP Investigators, Joëls M, Ophoff RA, Boks MP. The effect of childhood maltreatment and cannabis use on adult psychotic symptoms is modified by the COMT Val158Met polymorphism. Schizophr Res. 2013 Aug 15.

## Yale Penn EA: Genetics of Substance Dependence - United States

Our sample included a total of 2,379 European American (EA) subjects from a cohort of small nuclear families and unrelated individuals originally collected to study the genetics of drug (opioid or cocaine) or alcohol dependence (Gelernter et al., 2014). Subjects gave written informed consent as approved by the institutional review board at each site, and certificates of confidentiality were obtained from NIDA and NIAAA. Yale/Penn subjects were administered the Semi-Structured Assessment for Drug Dependence and Alcoholism (SSADDA) (Pierucci-Lagha et al, 2005) to derive DSM-V diagnoses of lifetime cannabis dependence and other major psychiatric traits. Lifetime cannabis use was assessed by the following question: “Have you ever used marijuana to feel good or high, or to feel more active or alert.” Age at first use, as well as a measure of frequency of during the period in the subject’s life in which they used the drug most heavily, was also assessed. A total of 1964 subjects for which both lifetime cannabis use and genotypes were available were used in this study.

**References**

Gelernter, J., Kranzler, H. R., Sherva, R., Almasy, L., Koesterer, R., Smith, A. H., ... & Farrer, L. A. (2014). Genome-wide association study of alcohol dependence: significant findings in African-and European-Americans including novel risk loci. *Molecular psychiatry*, *19*(1), 41-49.

Pierucci-Lagha, A., Gelernter, J., Feinn, R., Cubells, J. F., Pearson, D., Pollastri, A., ... & Kranzler, H. R. (2005). Diagnostic reliability of the Semi-structured Assessment for Drug Dependence and Alcoholism (SSADDA). *Drug and alcohol dependence*, *80*(3), 303-312.

# Replication samples

## RADAR: Research on Adolescent Development and Relationships – The Netherlands

The RADAR study (Research on Adolescent Development and Relationships) is longitudinal research project in the Netherlands and focuses on the development of interpersonal relationships, personality, and psychopathology, in a sample of adolescents and their families that were followed from approximately ages 13 to 19. Currently, there are 7 waves available (collected between 2006 and 2013), but the study is still ongoing. The RADAR study has a focus on delinquency development, therefore adolescents at risk for externalizing behavior were oversampled, which was determined by a having a T-score > 60 on the externalizing scale of the Teacher’s Report Form at age 12 (TRF; Achenbach, 1991; Verhulst, van der Ende, & Koot, 1997). In total, 497 adolescents were included in the study, of which 206 (41.45%) were at high risk for externalizing behavior (for more information on the sample see Creemers et al., 2015). The study was approved by the medical ethical committee of Utrecht University. Families received 100 Euros for each home visit. The data on lifetime cannabis use were collected every year as part of home assessments, during which research assistants visited the adolescents and their families (wave 1-7). Using self-report questionnaires, adolescents were asked to indicate how often they had used hash or weed in the past 12 months. Answer categories were 0 times, 1 time, 2 times, 3 times, 4 times, 5 times, 6 times, 7 times, 8 times, 9 times, 10 times, 11-19 times, 20-39 times, 40 times or more. Additionally, adolescents were asked how old they were when they had used weed or hash for the first time, but only in the last wave (wave 7). For the present analyses, lifetime cannabis use was calculated based on all annual assessments.

In wave 5, 416 adolescents provided genotype data. For the current study, valid genotype and phenotype data were available for 338 adolescents. The mean age at wave 7 was 19.54 (SD = 0.78, range = 17 – 22).

References:

Achenbach, T. M. (1991). Manual for the youth self report and 1991 profiles. Burlington: Department of Psychiatry, University of Vermont.

Creemers, H. E., Buil, J. M., van Lier, P. A.C., Keijsers, L., Meeus, W., Koot, H. M., & Huizink, A. C. (2015). Early onset of cannabis use: Does personality modify the relation with changes in perceived parental involvement? *Drug and Alcohol Dependence, 146,* 61-67.

Verhulst, F. C., van der Ende, J., & Koot, H. M. (1997). Handleiding voor de Youth-Self-Report [Manual for the Youth Self-Report]. Rotterdam, The Netherlands: Afdeling jeugdpsychiatrie, Sophia Kinderziekenhuis/Academisch Ziekenhuis Rotterdam/Erasmus Universiteit Rotterdam.

## The Saguenay Youth Study (SYS)

## The Saguenay Youth Study (SYS) is a population-based study of adolescents and their middle-aged parents1. It is aimed at investigating the etiology, early stages and trans-generational trajectories of common cardio-metabolic and brain diseases. The SYS was designed as a two-generational cohort; it includes 1,029 adolescents and their 962 parents. The cohort was recruited via 12- to 18-year old adolescents attending high schools in the Saguenay Lac-Saint-Jean region of Quebec (Canada). Half of the adolescents were exposed prenatally to maternal cigarette smoking. The cohort is family-based (n=481 families), including only adolescents who have one or more siblings of similar age (i.e., 12 to 18 years) and both biological parents of the French-Canadian origin born in the region. The data collection occurred in two waves. Wave 1 (2003-2012) involved the recruitment and complete assessment of all 1,028 adolescents, as well as a partial (‘soft’) assessment of 962 parents. Wave 2 (2012-2015) involved the complete assessment of a subset of the parents (n=664). In Wave 2, parents answered a series of questions about their drug use; this questionnaire was based on the European School Survey Project on Alcohol and Other Drugs (http://www.espad.org/). The GWAS was based on answering (Yes/No) the following question: “Have you ever used marijuana (grass, pot) or hashish (hash, hash oil)?” with the following answer categories: 1=11 or less 2= 12 3=13 4=14 5=15 6=16 7=17 8=18 and more 9= prefer to not answer. Of the total sample (N=551), 262 individuals did not initiate cannabis use, 189 individuals initiated cannabis use at an age between 12 and 17 years and 100 individuals said they did so at 18 years of later. The age at initiation for this last group is set on 18 years old in order to allow us to use the full sample.

##

References:

## Paus, T., Pausova, Z., Abrahamowicz, M., Gaudet, D., Leonard, G., Pike, G.B., & Richer, L. (2015). Saguenay Youth Study: A multi-generational approach to studying virtual trajectories of the brain and cardio-metabolic health. Developmental cognitive neuroscience, 11; 137-144.

##

## TwinsUK – United Kingdom

TwinsUK cohort is a longitudinal population-based adult registry, which is not enriched for any particular disease or trait and is representative of the British general population of Caucasian ethnicity.  The twins were volunteers recruited through a national media campaign ([www.twins.ac.uk](http://www.twins.ac.uk/)).  Questions pertaining to cannabis use was asked in a lifestyle questionnaire in autumn 2009, questionnaire 18.  The question asked was: ”Have you ever smoked cannabis for recreational or medicinal purposes?”  The answer categories were “Yes” or “No”.  From the total number of question respondents, 664 individuals said they had smoked cannabis, and 3841 said they had never smoked cannabis.  Of these individuals, 1000 genome imputed whole genome genotyping data was available for 2,137 individuals of which 2,078 could be used for genetic analysis.  The individuals included in this study had an average of 58 years, ranging from 18-86 years.

## Yale Penn AA: Genetics of Substance Dependence – United States

Our sample included a total of 3,318 African American (AA) subjects from a cohort of small nuclear families and unrelated individuals originally collected to study the genetics of drug (opioid or cocaine) or alcohol dependence (Gelernter et al., 2014). Subjects gave written informed consent as approved by the institutional review board at each site, and certificates of confidentiality were obtained from NIDA and NIAAA. Yale/Penn subjects were administered the Semi-Structured Assessment for Drug Dependence and Alcoholism (SSADDA) (Pierucci-Lagha et al., 2005) to derive DSM-V diagnoses of lifetime cannabis dependence and other major psychiatric traits. Lifetime cannabis use was assessed by the following question: “Have you ever used marijuana to feel good or high, or to feel more active or alert.” Age at first use, as well as a measure of frequency of during the period in the subject’s life in which they used the drug most heavily, was also assessed. A total of 2,660 subjects for which both lifetime cannabis use and genotypes were available were used in this study.

**References**

Gelernter, J., Kranzler, H. R., Sherva, R., Almasy, L., Koesterer, R., Smith, A. H., ... & Farrer, L. A. (2014). Genome-wide association study of alcohol dependence: significant findings in African-and European-Americans including novel risk loci. *Molecular psychiatry*, *19*(1), 41-49.

Pierucci-Lagha, A., Gelernter, J., Feinn, R., Cubells, J. F., Pearson, D., Pollastri, A., ... & Kranzler, H. R. (2005). Diagnostic reliability of the Semi-structured Assessment for Drug Dependence and Alcoholism (SSADDA). *Drug and alcohol dependence*, *80*(3), 303-312.
