# Supplementary material for: Genome-wide association study of lifetime cannabis use based on a large meta-analytic sample of 32 330 subjects from the International Cannabis Consortium
Source: Transl Psychiatry. 2016 Mar 29;6(3):e769–. doi: 10.1038/tp.2016.36 (PMC4872459; doi:10.1038/tp.2016.36)
Supplement: Supplementary Information 2 [file tp201636x2.doc]

# SNP based heritability estimates

The proportion of phenotypic variance explained by genome-wide SNPs was estimated using the density estimation (DE) method developed by So et al. . The DE method evaluates the difference between the observed distribution of test statistics in the meta-analysis and the corresponding null distribution to estimate the distribution of effects genome-wide. Estimated effect sizes are adjusted for “winner’s curse”, and transformed to an estimate of the proportion of variance explained. Prior to estimation, SNPs present in at least 25% of the meta-analysis samples were pruned for LD, r^2^ >0 .15. Pruning was performed using Priority Pruner with 1000 Genomes reference data to preferentially select SNPs with the largest observed sample size in the meta-analysis, resulting in 200,003 SNPs retained for estimation. Following Walters , 200 wild bootstrap replicates were used to adjust for potential bias from asymmetric effect size distributions, and Monte Carlo p-values were used to test the observed estimate compared to 1000 estimates under the simulated null hypothesis of no variance explained by genome-wide SNPs.

# Estimating the genetic covariation between lifetime cannabis use and cigarette smoking

LD Score regression estimates the variance explained by SNPs based on the expected relationship between linkage disequilibrium (LD) and strength of association under a polygenic model and an extension of this method can be used to estimate the genetic covariation between different traits . Here, we estimated the degree of genetic overlap between lifetime cannabis use and lifetime cigarette smoking, using the summary statistics from our meta-analysis and a meta-analysis on ever versus never smoking of the Tobacco and Genetics Consortium . Estimating the genetic correlation from summary statistics is based on the fact that the estimated GWA effect-size for a SNP incorporates the effects of all SNPs in LD with that SNP. The genetic covariance is estimated using the slope from the regression of the product of z-scores from the two GWAS studies on the LD Score .

For this analysis, we included the intersection of SNPs present in all ICC contributing cohorts and - as suggested by Bulik-Sullivan et al. – the SNPs present in the HapMap 3 reference set (N_SNPs_=433,918). Accordingly, the sample size for this analysis was set to N=32,330. For the ever vs. never smoking analysis the sample size was set to N=74,053. LD scores were computed for each SNP based on the LD observed in the 1000 genomes references data.

# References

Bulik-Sullivan, B., Finucane, H. K., Anttila, V., Gusev, A., Day, F. R., ReproGen Consortium, . . . Neale, B. M. (2015). An atlas of genetic correlations across human diseases and traits.

Edlund, C. K., Anker, M., Schumacher, F. R., Gauderman, W. J., & Conti, D. V. (2014). Priority Pruner (version 0.1.1).

So, H. C., Li, M., & Sham, P. C. (2011). Uncovering the total heritability explained by all true susceptibility variants in a genome-wide association study. *Genet Epidemiol, 35*(6), 447-456. doi:10.1002/gepi.20593

Tobacco and Genetics Consortium. (2010). Genome-wide meta-analyses identify multiple loci associated with smoking behavior. *Nat Genet, 42*(5), 441-447. doi:10.1038/ng.571

Walters, R. K. (2014). *Estimating variance explained by all DNA loci in a genome-wide meta-analysis (Doctoral dissertation).* .
