# Supplementary material for: Genome-wide association study of lifetime cannabis use based on a large meta-analytic sample of 32 330 subjects from the International Cannabis Consortium
Source: Transl Psychiatry. 2016 Mar 29;6(3):e769–. doi: 10.1038/tp.2016.36 (PMC4872459; doi:10.1038/tp.2016.36)
Supplement: Supplementary Information 3 [file tp201636x3.docx]

**ICC – International Cannabis Consortium**

Analysis plan for GWAS on cannabis use phenotypes

Last updated: August 12^th^ 2013

This document provides the instructions for the data preparation and GWA analyses on cannabis use phenotypes in each of the participating cohorts. Standardisation of the procedures is very important as it will increase the precision of the meta-analyses across all samples of the consortium.

*Study aim:*

The International Cannabis Consortium has been created to combine the results of multiple genome-wide association studies of cannabis use (lifetime use, age at initiation, regular/frequency of use) in meta-analyses in order to increase the probability of detection of genetic variants associated with individual differences in liability to cannabis use. At this first discovery phase about ~27,000 individuals will be included.

**Deadline**

Please upload the GWAS results of your sample before: 1 August 2013

**Contact details:**

Questions about this document can be directed to: Jacqueline Vink, [j.m.vink@vu.nl](mailto:j.m.vink@vu.nl)

**The working group**:

Dr. Jacqueline Vink [jm.vink@vu.nl](mailto:jm.vink@vu.nl) (PI)

Camelia Minica MSc. [c.c.minica@vu.nl](mailto:c.c.minica@vu.nl)

Dr. Karin Verweij [karin.verweij@vu.nl](mailto:karin.verweij@vu.nl)

Prof. Dr. Eske Derks [e.m.derks@amc.uva.nl](mailto:e.m.derks@amc.uva.nl)

Dr. Nathan Gillespie [ngillespie@vcu.edu](mailto:ngillespie@vcu.edu)

Sven Stringer MSc. svenstringer@gmail.com

**Content of this document:**

-Instructions for phenotype and covariate coding

-Instructions for genotype handling

-Instructions for association tests

-Instructions for uploading data

-Meta-analyses

-Authorship

-Participating cohorts

-Data overview

**Instructions for phenotypes and covariate coding**

*Inclusion*

We propose to limit the analyses to subjects from European ancestry (based on both self-report and principal components). Please let us know if you have a large group of individuals of non-European ancestry.

*Cannabis use phenotypes*

Based on the available data we would like to start the first round of analyses with:

**- Lifetime cannabis use** (binary variable). Please code 2=ever and 1=never. Note that we will only include individuals of 18 years and older for this analysis.

*Covariates - Please use these variablse as a covariate in your GWAS analysis:*

- **Age** at the time of the phenotypic assessment (in years since birth).
- **Sex** coded as 1=male, 2=female.
- **Birth cohort** (to correct for cohort effects). Please recode year of birth in groups spanning 20 year-periods and make a dummy variable for each of the cohorts except the reference group. Use the lowest birth cohort as the reference group.

So for example: If the birth range in your cohort is 1940 to 1992, please make 3 birthcohorts, namely 1940-1959, 1960-1979 and 1980-2000. The lowest birth cohort (1940-1959) will be the reference group, so we need two dummy variables:

BirthCohortDummyA is ‘1’ if the subject is born in 1960-1979 and ‘0’ otherwise.

BirthCohortDummyB is ‘1’ if the subject is born in 1980-2000 and ‘0’ otherwise.

Include the dummy variables as covariates in the GWA analyses.

Information about dummy coding if you will use Plink can be found here:

<http://pngu.mgh.harvard.edu/~purcell/plink/dataman.shtml#wrtcov>

- **Population structure**. Please use the first four principal components to correct for population structure in your sample. If necessary add study-specific covariates such as study site or batch effects

Missing values (in phenotypes) should be coded -9 for Plink.

**Instructions for genotype handling**

*Pre imputation QC*

We assume genotyping data have already gone through extensive quality control Typically, studies have excluded SNPs from further analysis (or imputation) with:

- Minor allele frequency <1%
- Call rate <95% or <99%
- HWE p < 1e-6
- Known to have evidence of poor clustering on visual inspection of intensity plots

Typically, studies have removed subjects that have:

- Low overall call rates (< 95%)
- Excess autosomal heterozygosity (indicating genotyping errors)
- Duplicate samples
- Unintended 1^st^ or 2^nd^ degree relatives in case of a sample of unrelated individuals
- Wrong gender (excessive X-chromosome homozygosity in males)
- XXY’s etc.

*Imputation*

Reference set used for imputation – **1000 Genomes release March 2012**. We prefer to use dosage data. Please filter out SNPs with poor imputation quality (r2_hat < 0.3 or proper_info < 0.4, depending on software used).

*Software for imputation:*

IMPUTE, MACH or other

If you would like to use another reference set for imputation, please contact Jacqueline Vink ([j.m.vink@vu.nl](mailto:j.m.vink@vu.nl)) to discuss the options.

Note: more stringent filtering may be performed when we run the meta-analysis.

Please provide us with an overview of your QC procedure by completing file “**overview_genotype_phenotype_CCI.xlsx**” and email it to [jm.vink@vu.nl](mailto:jm.vink@vu.nl)

**Instructions for association tests in Plink**

*Association tests*

- All 22 autosomes
- Use dosage analysis to account for genotype imputation uncertainty. If dosage are not available please contact Jacqueline Vink ([jm.vink@vu.nl](mailto:jm.vink@vu.nl))
- When the sample includes family members the family structure should be taken into account. When the sample includes MZ twins please choose 1 individual per twin pair. To analyse family-based samples, please correct for relatedness (PLINK --family option), see command lines below.
- Given there is no evidence for non-additive genetic variance in cannabis use phenotypes, we will only run additive association tests: PHENOTYPE = b0 + b1*SNP + b2*sex + b3*age + b4*.... [include all covariates]
- For lifetime use we will run a logistic regression in Plink. Please see below for the specific command lines.

*Type of software to use:*

- Please use **PLINK** (see commands below).

*Command lines in Plink:*

Important tip: please use the same order of samples in the fam file as it appears in the dosage data!

**Lifetime cannabis use** (ever/never use – binary variable). Please code 2=ever and 1=never. Note that we will only include individuals of 18 years and older for this analysis. Please include covariates age, sex, birth cohort, population structure (see above).

LOGISTIC REGRESSION

*#analysis -  family data:*

plink --dosage **Name_genotypeFile.gz Zin noheader skip0=? skip1=? format=?** --fam Name_phenotypeFile --family --logistic --covar Name_covariatesFile --out Name_outputFile

*#analysis - unrelated individuals:*

plink --dosage **Name_genotypeFile.gz Zin noheader skip0=? skip1=? format=?** --fam Name_phenotypeFile --logistic --covar Name_covariatesFile --out Name_outputFile

Please note: This example specifies (shown in bold in the command line) that the dosage data are compressed (Zin) and that there are no headers available (noheader). Please see the Plink manual  - the Dosage data section: <http://pngu.mgh.harvard.edu/~purcell/plink/dosage.shtml#assoc> – for **OPTIONS** to modify the expected  format of the input dosage data .

**Instructions on the format of the input files for the Meta-analysis**

Data format - Please strictly adhere to the data format as specified below in this table!

| Column: | Variable Name: | Description: | Type/Format |
| --- | --- | --- | --- |
| 1 | SNP | CHR_BP name if 1000G was used for imputation | STRING |
| 2 | CHR | Chromosome | NUMERIC, no decimals |
| 3 | POS | Position | NUMERIC, no decimals |
| 4 | EFF_ALL | Effect or Coded allele, for which the linear regression effect is reported (A/T/G/C). In PLINK output this is A1 | STRING |
| 5 | NONEFF_ALL | Non-effect or Non-coded allele (A/T/G/C).  In PLINK output this is A2 | STRING |
| 6 | STRAND | Strand, on which the alleles are reported (‘+’/ ‘-‘). After imputation, this is typically the + strand. | STRING |
| 7 | OR | Odds Ratio (from logistic regression) | NUMERIC, 4 decimals |
| 8 | SE | Standard error of effect of additive test. | NUMERIC, 4 decimals |
| 9 | P | P-value of additive test | NUMERIC, scientific notation, e.g. 1.02E-06 |
| 10 | AF_COD | Allele Frequency, of the coded allele specified in column 4. In Plink output this is FRQ. | NUMERIC, 4 decimals |
| 11 | MAF | Minor Allele Frequency | NUMERIC, 4 decimals |
| 12 | HWE | HWE p-value | NUMERIC, scientific notation, e.g. 1.02E-03 |
| 13 | IMP | Whether a SNP was observed (=0) or imputed (=1) | NUMERIC, no decimals |
| 14 | INFO | Imputation quality for imputed SNPs, set to 1 if the SNP was directly genotyped. Report R2hat or proper_info depending on software used. In PLINK output this is INFO. | NUMERIC, 4 decimals |
| 15 | INFO_TYPE | Measure used to report imputation quality:  0=R2hat,  1=proper_info,  2=info from PLINK | NUMERIC, no decimals |
| 16 | N_EFF | Sample size (number of individuals with genotype (imputed or direct) and phenotype data. Note that this can differ per SNP) | NUMERIC, no decimals |

- Please save your file with results as plain space-delimited text file.
- Missing values should be coded as -999
- Please provide the variable names in the first row of the file. Please use the exact same phrasing.
- Please keep the order of the variables as requested above.

Header line: SNPID CHR POS EFF_ALL NONEFF_ALL STRAND OR SE_ADD P_ADD AF_COD MAF HWE IMP INFO INFO_TYPE N_EFF
