# Supplementary material for: Genome-wide association study of lifetime cannabis use based on a large meta-analytic sample of 32 330 subjects from the International Cannabis Consortium
Source: Transl Psychiatry. 2016 Mar 29;6(3):e769–. doi: 10.1038/tp.2016.36 (PMC4872459; doi:10.1038/tp.2016.36)
Supplement: Supplementary Table 3 [file tp201636x6.docx]

**Supplemental Table S3. Replication results of the top 10 independent SNPs (R^2^<0.1) in the European and African-American samples.**

|  |  |  |  |  | **Europeans** |  | **African-Americans** |  |
| --- | --- | --- | --- | --- | --- | --- | --- | --- |
| **SNP** | **Chr** | **BP (hg19)** | **A1** | **A2** | **beta (s.e.)** | **p-value** | **beta (s.e.)** | **p-value** |
| rs4984460 | 15 | 96424399 | T | G | -.08 (.07) | .26 | .05 (.07) | .44 |
| rs2099149 | 12 | 30479358 | T | G | -.23 (.14) | .12 | .02 (.07) | .72 |
| rs7675351 | 4 | 141218757 | A | C | -.12 (.26) | .64 | -.13 (.08) | .10 |
| rs4471463  rs7107977 | 11  11 | 112983595  915764 | T  A | C  G | -.005 (.06)  .13 (.12) | .94  .26 | .02 (.07)  .09 (.07) | .70  .22 |
| rs58691539 | 2 | 52753909 | T | G | NA* | NA | -.11 (.08) | .18 |
| rs2033867  rs35053471 | 2  3 | 175188281  47124761 | A  A | G  T | .03(.20)  .03(.07) | .85  .66 | .12 (.18)  . 11(.09) | .49  .25 |
| rs12518098 | 5 | 60864467 | C | G | -.04(.11) | .72 | .07 (.14) | .61 |
| rs73067624 | 1 | 196333461 | T | C | .27(.19) | .15 | -.03 (.10) | .72 |

*SNP not available in the sample

Note: Association results are included if present in at least 1 sample
