# Supplementary material for: Genome-wide association study of lifetime cannabis use based on a large meta-analytic sample of 32 330 subjects from the International Cannabis Consortium
Source: Transl Psychiatry. 2016 Mar 29;6(3):e769–. doi: 10.1038/tp.2016.36 (PMC4872459; doi:10.1038/tp.2016.36)

Supplementary Figures

Contents

[Figure S1a-b. The Manhattan (a) and the quantile-quantile plots (b) based on the meta-analytic discovery sample 1](#_Toc428533290)

[Figure S2a-m. Manhattan plots for lifetime cannabis use per sample 2](#_Toc428533291)

[Figure S3a-m. Quantile-quantile plots for lifetime cannabis use per sample 10](#_Toc428533292)

[Figure S4a-j. Regional association plot showing signal around top SNPs 23](#_Toc428533293)

[Figure S5. Forest plots key SNPs in the 5 significant regions 29](#_Toc428533294)

[Figure S6a-e. Regional plots around the 5 significant regions 38](#_Toc428533295)

# Figure S1a-b. The Manhattan (a) and the quantile-quantile plots (b) based on the meta-analytic discovery sample

In the Manhattan plot, the y-axis is the strength of association(-log_10_(P)) and the x-axis shows the chromosomal position. The blue line indicates suggestive significance level (P-values < 10^-5^) while the red line indicates genome-wide significance level (P-values < 5x10^-8^).

a.


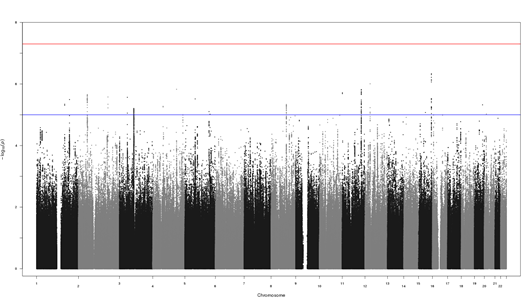


b.


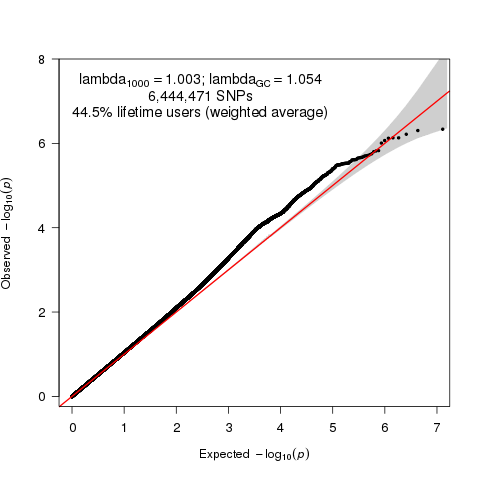


# Figure S2a-m. Manhattan plots for lifetime cannabis use per sample

a. Avon Longitudinal Study of Parents and Children (ALSPAC)


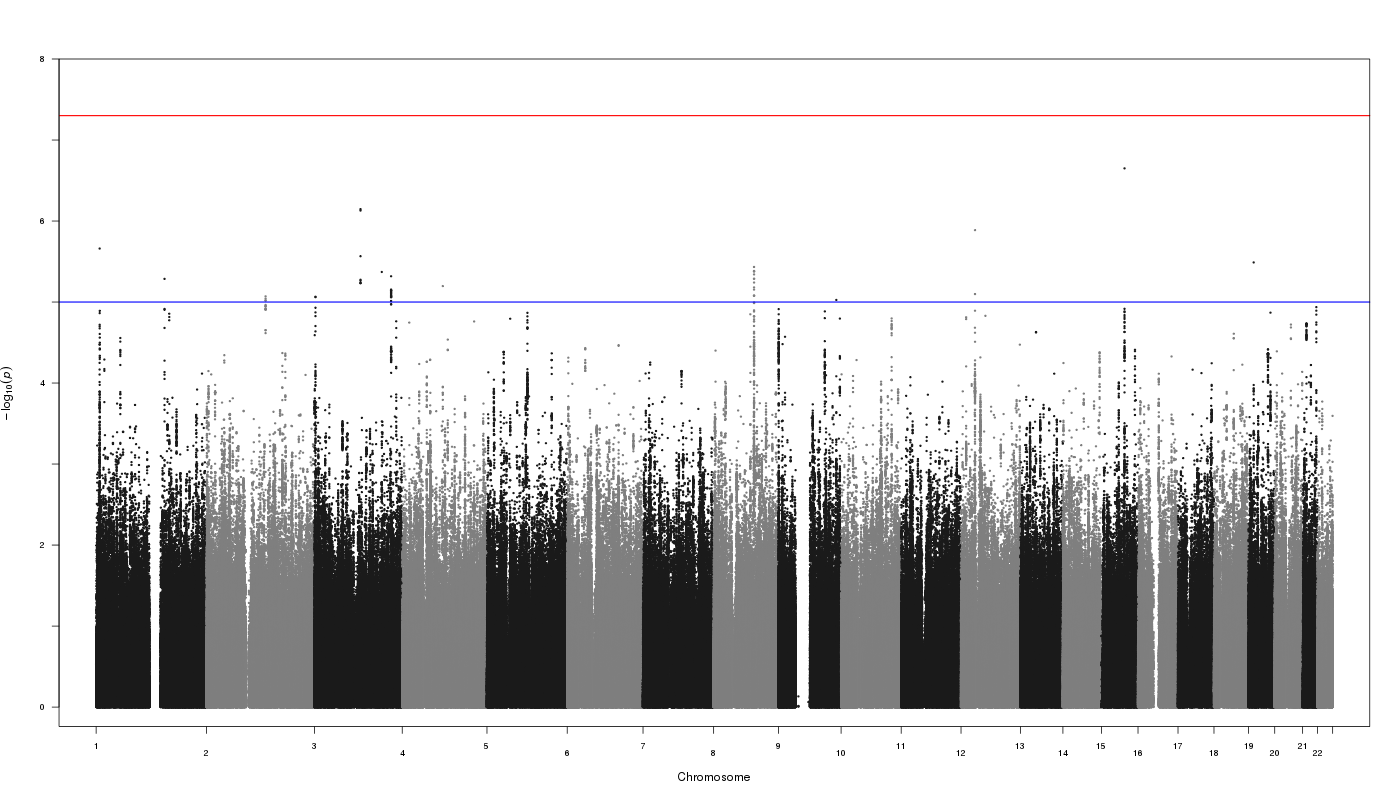


b. Brisbane Longitudinal Twin Study (BLTS)


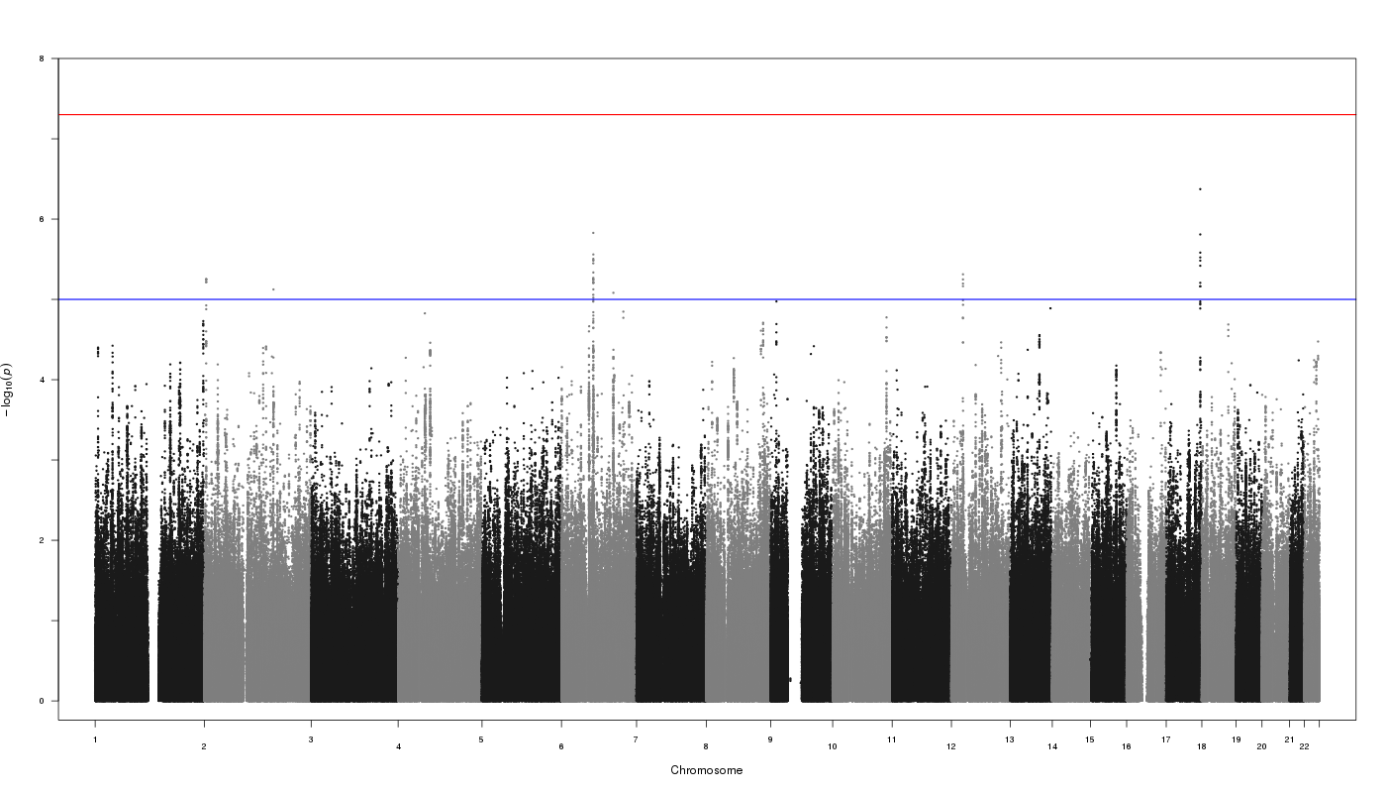


c. Center for Antisocial Drug Dependence (CADD)


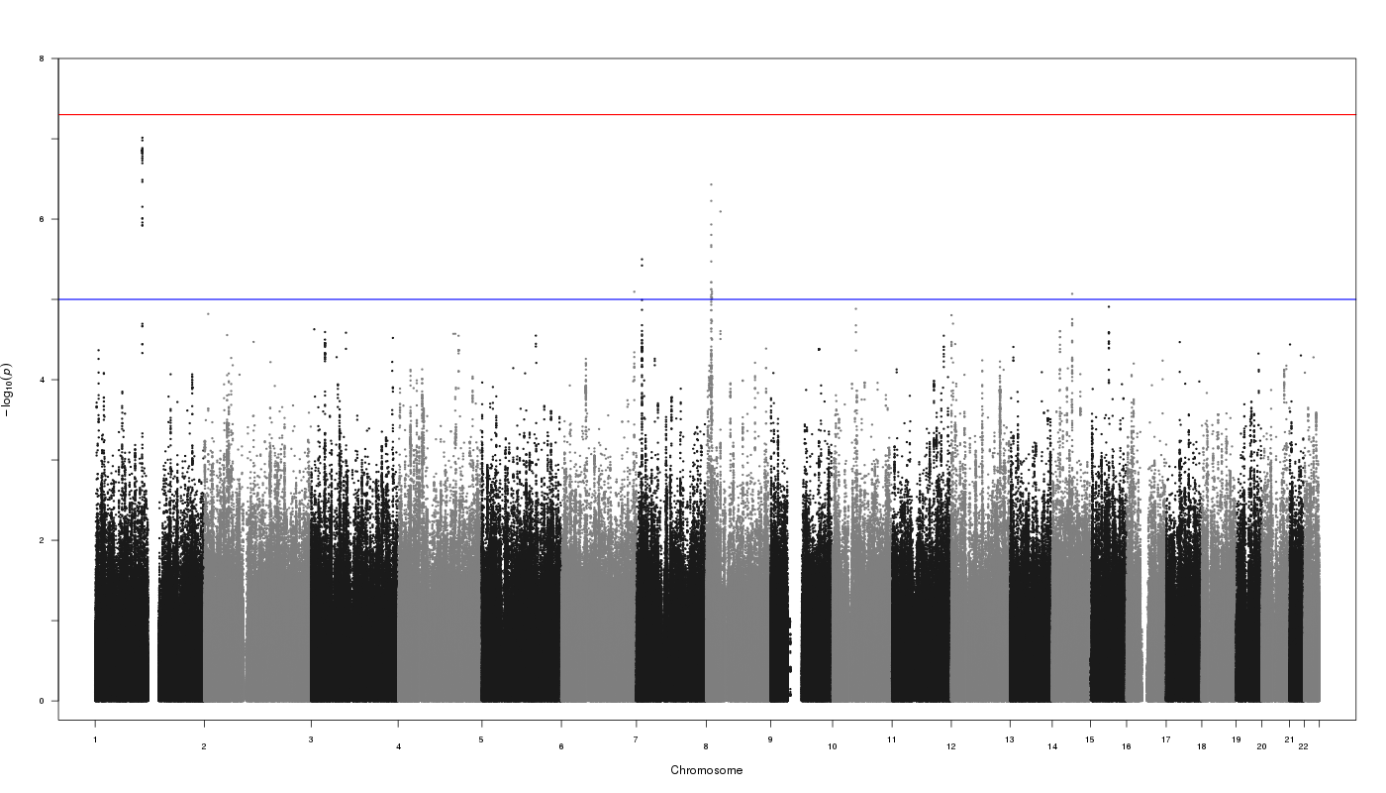


d. Estonian Genome Center University of Tartu -1 (EGCUT1)


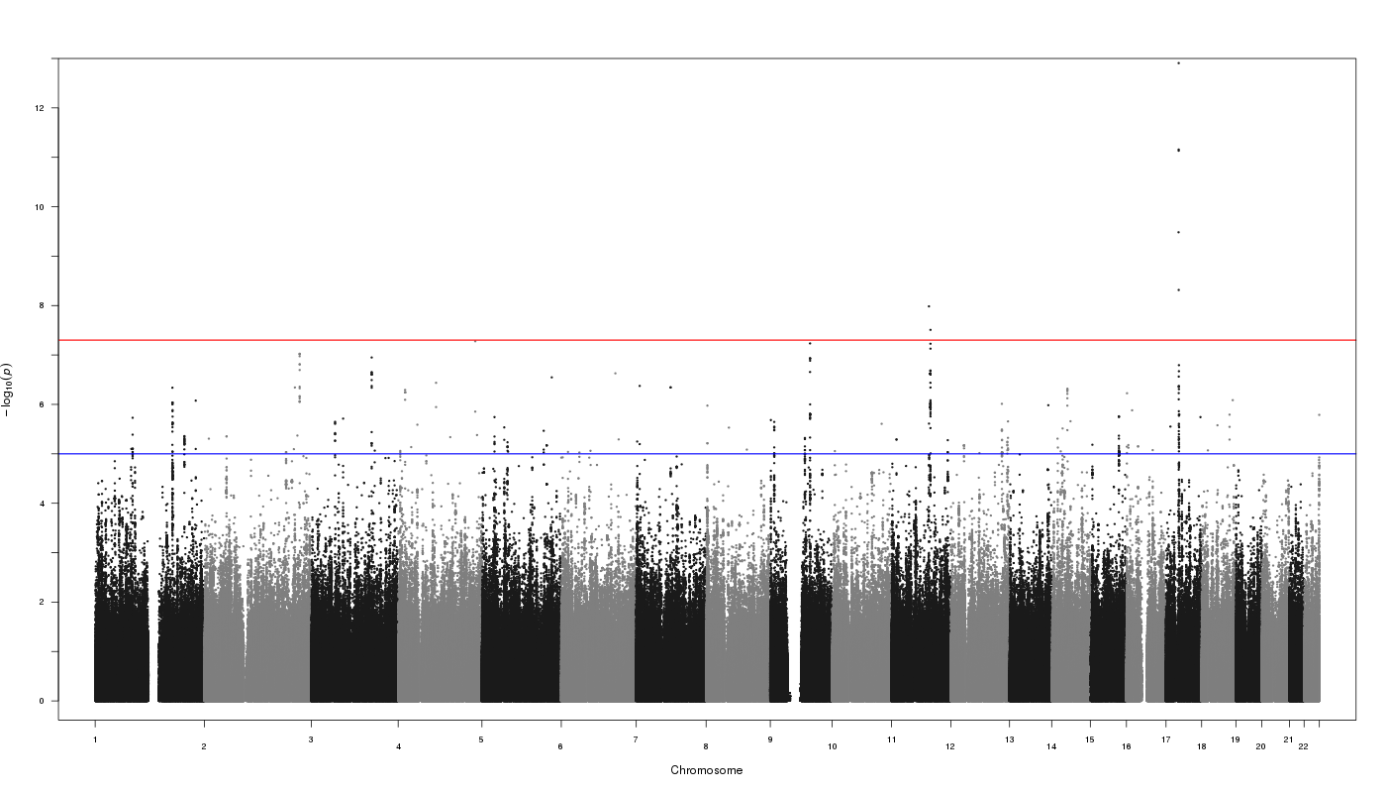


e. Estonian Genome Center University of Tartu -2 (EGCUT2)


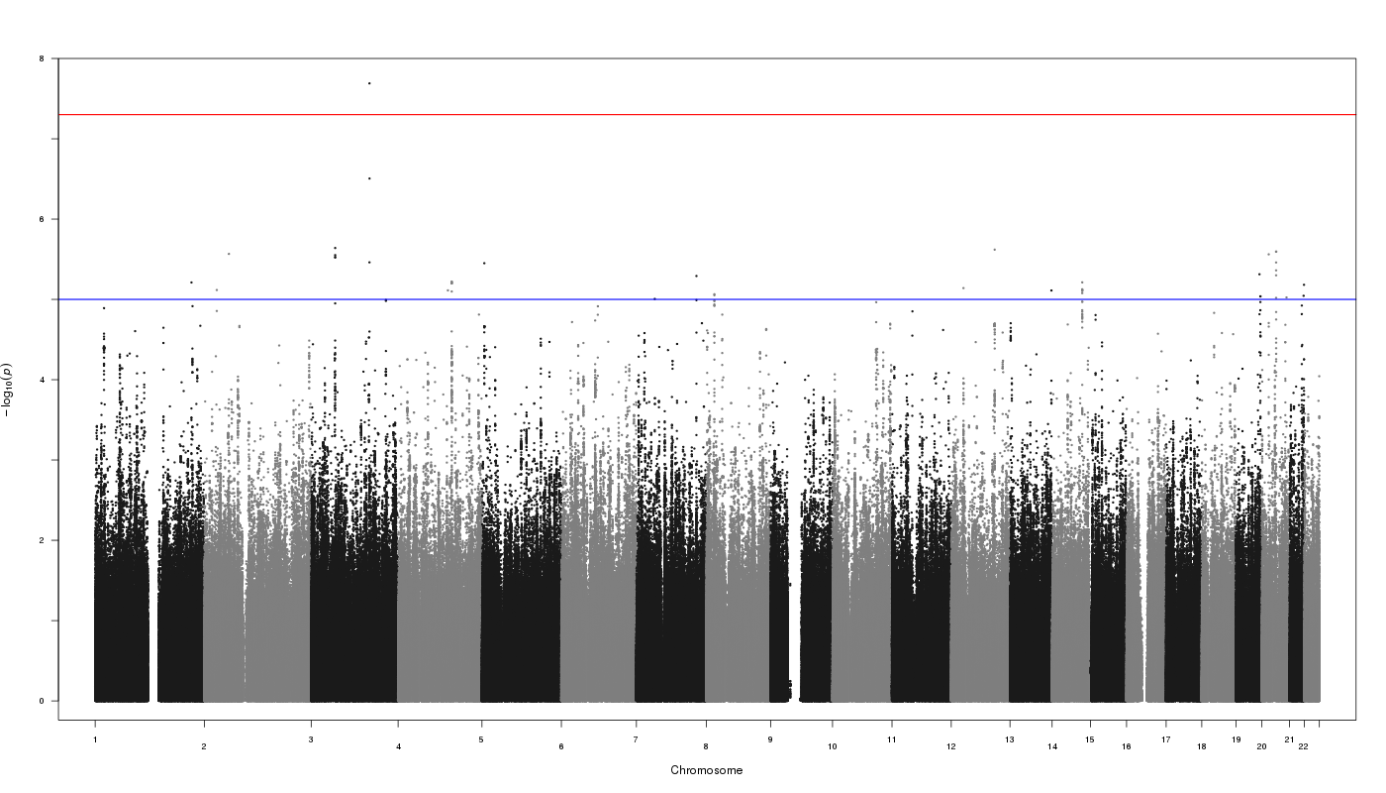


f. Finnish Twin Cohort (FinnTwin12 & FinnTwin16)


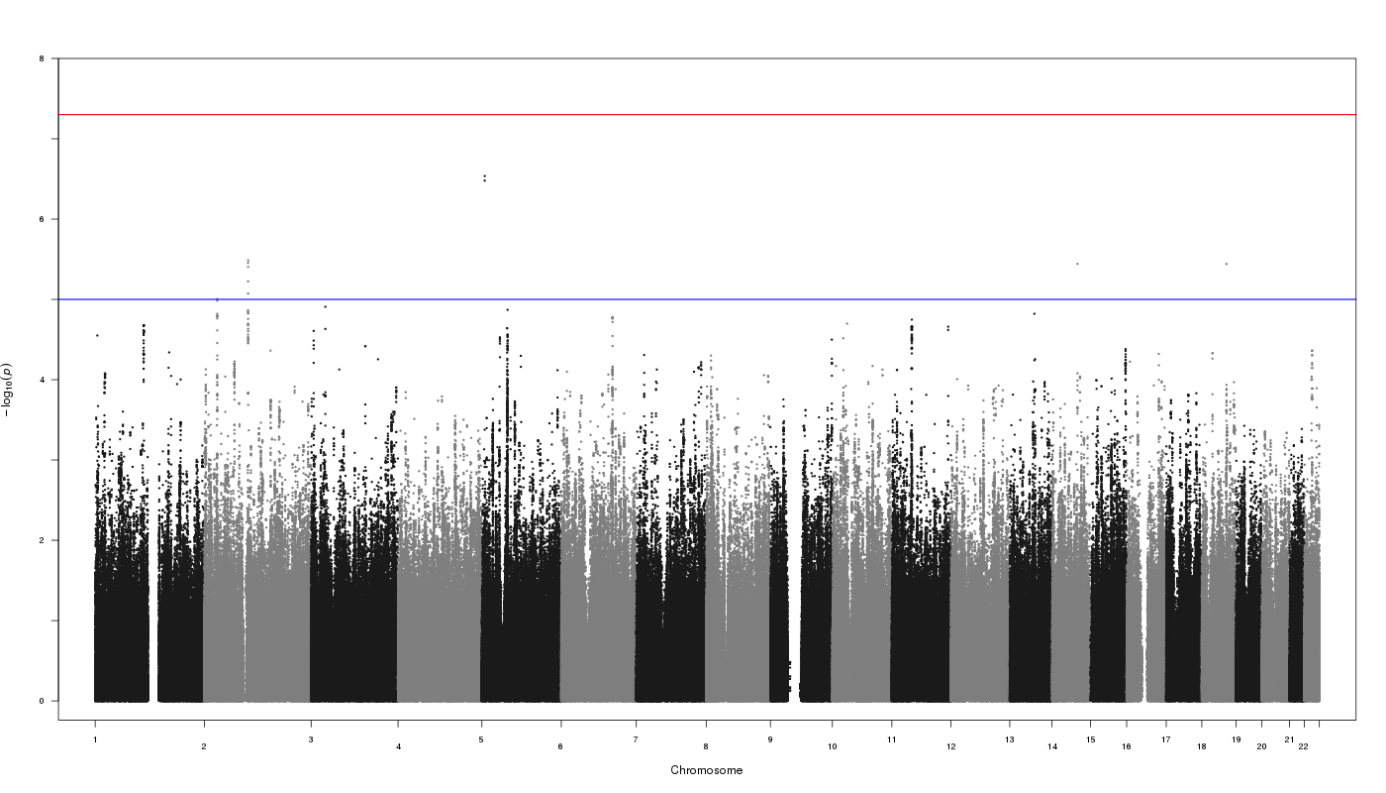


g. Genetics of Substance Dependence - European American (Yale Penn EA)


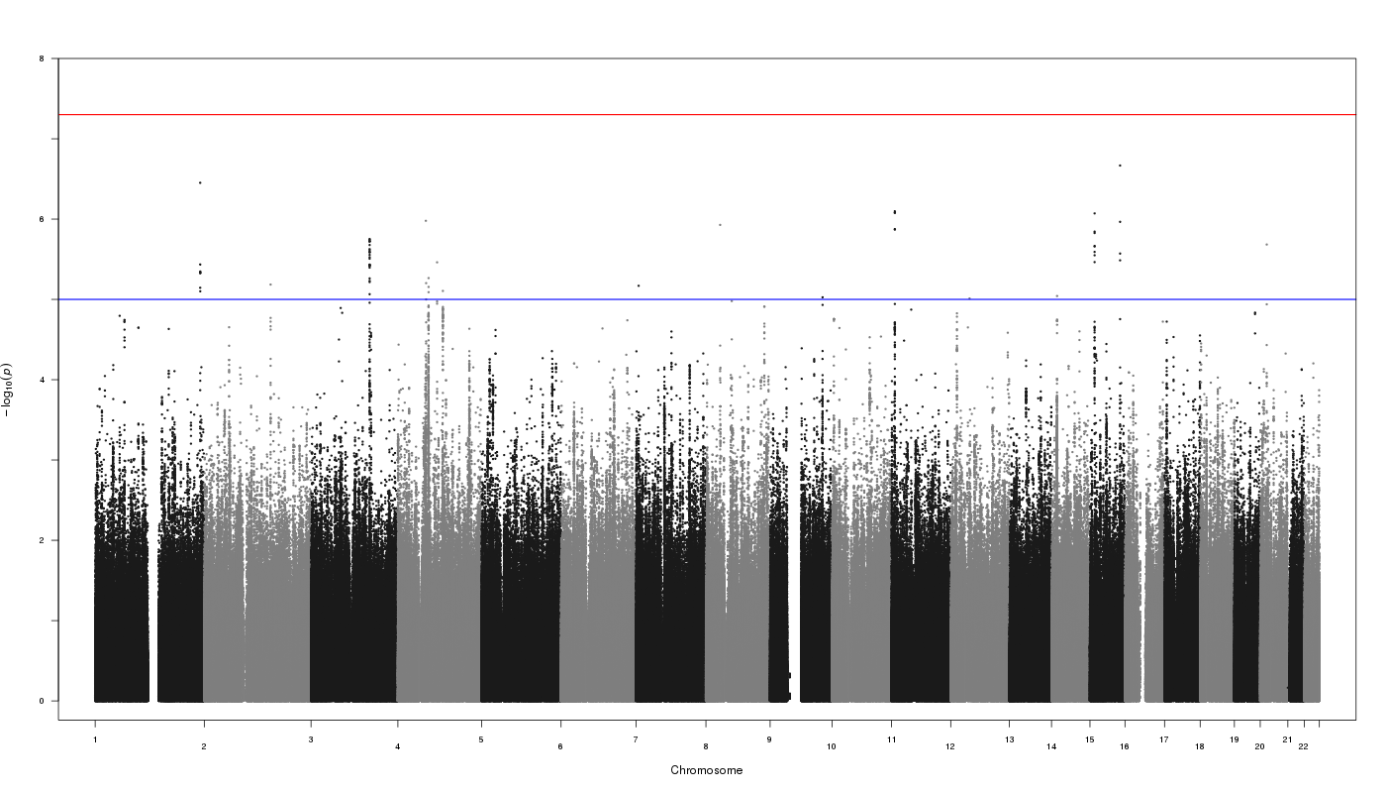


h. Hospital Universitari Vall d'Hebron – Barcelona (HUVH-Barcelona)


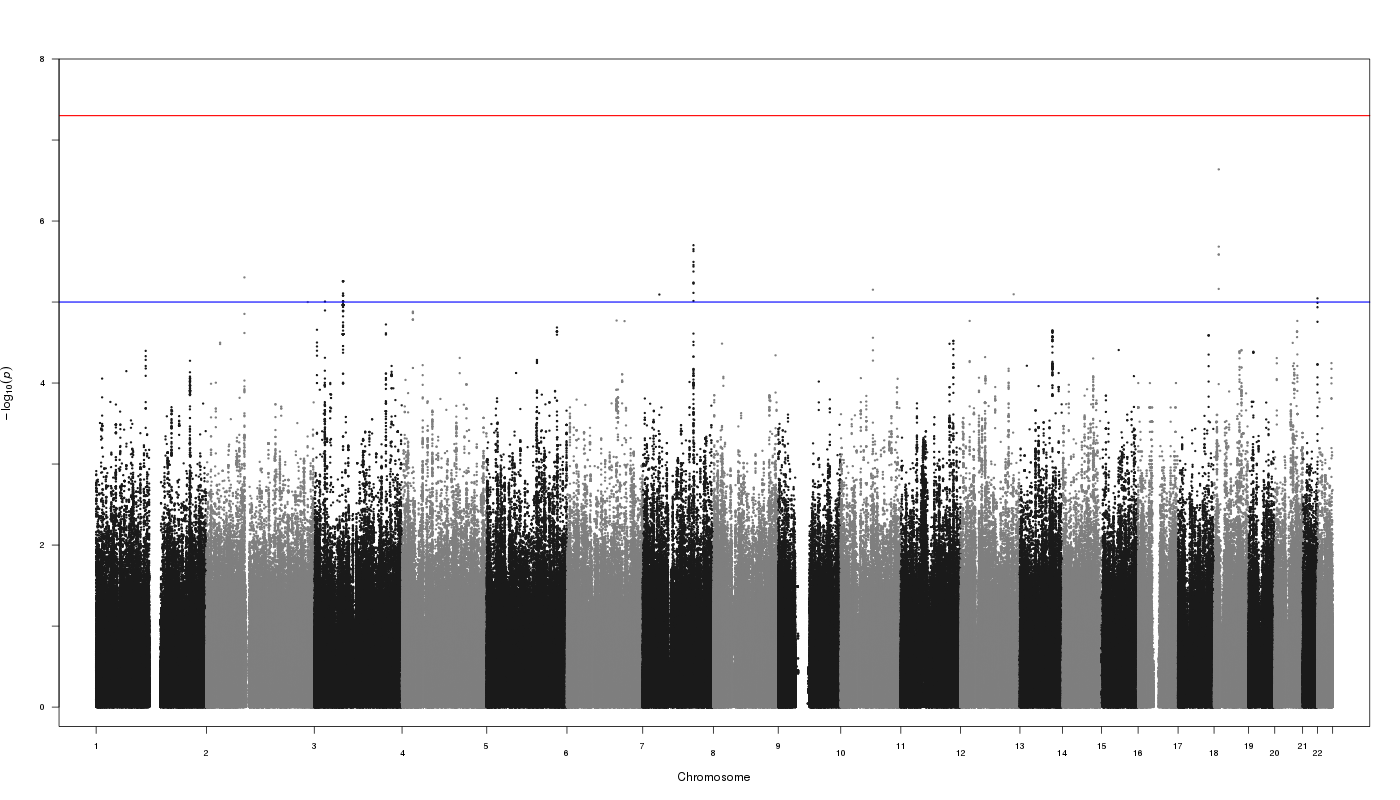


i. Minnesota Center for Twin and Family Research (MCTFR)


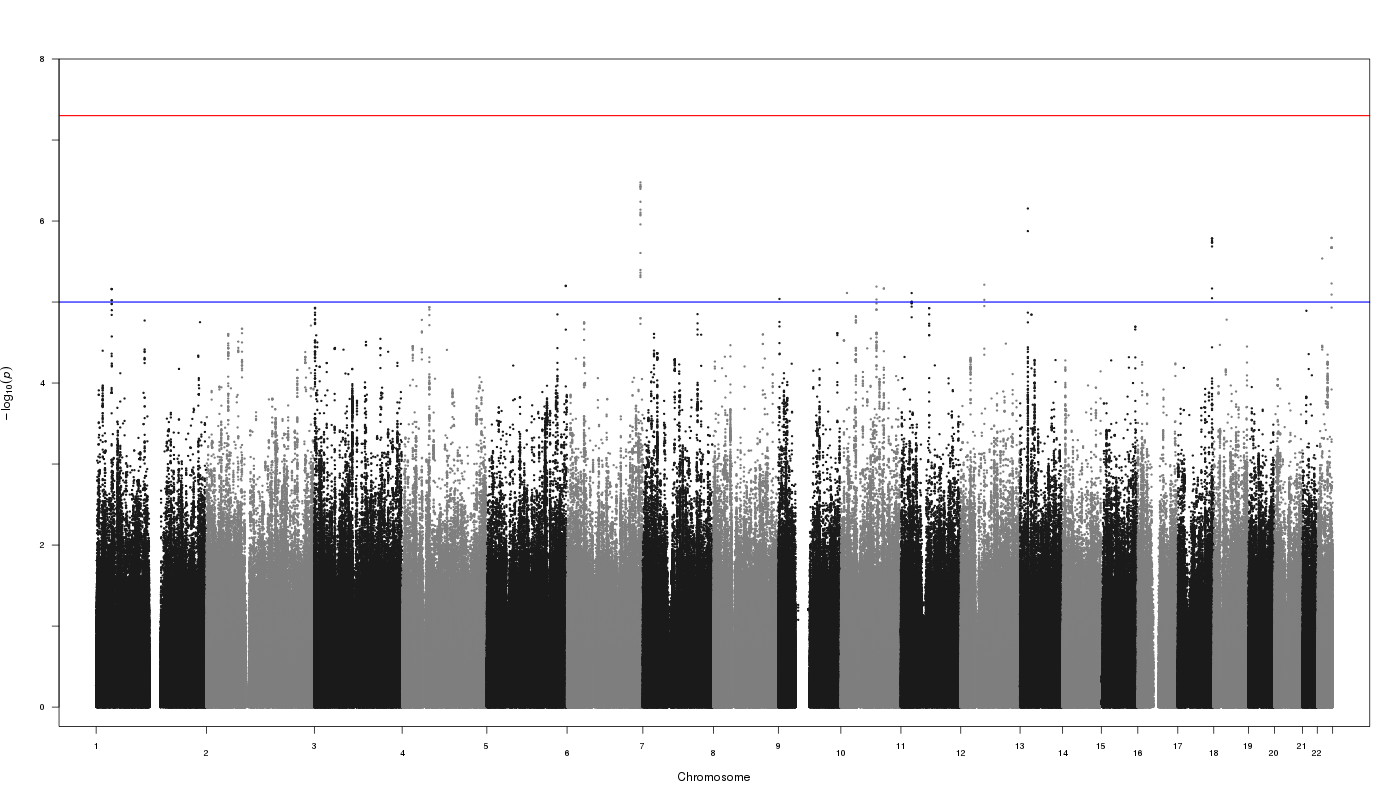


j. Netherlands Twin Register (NTR)


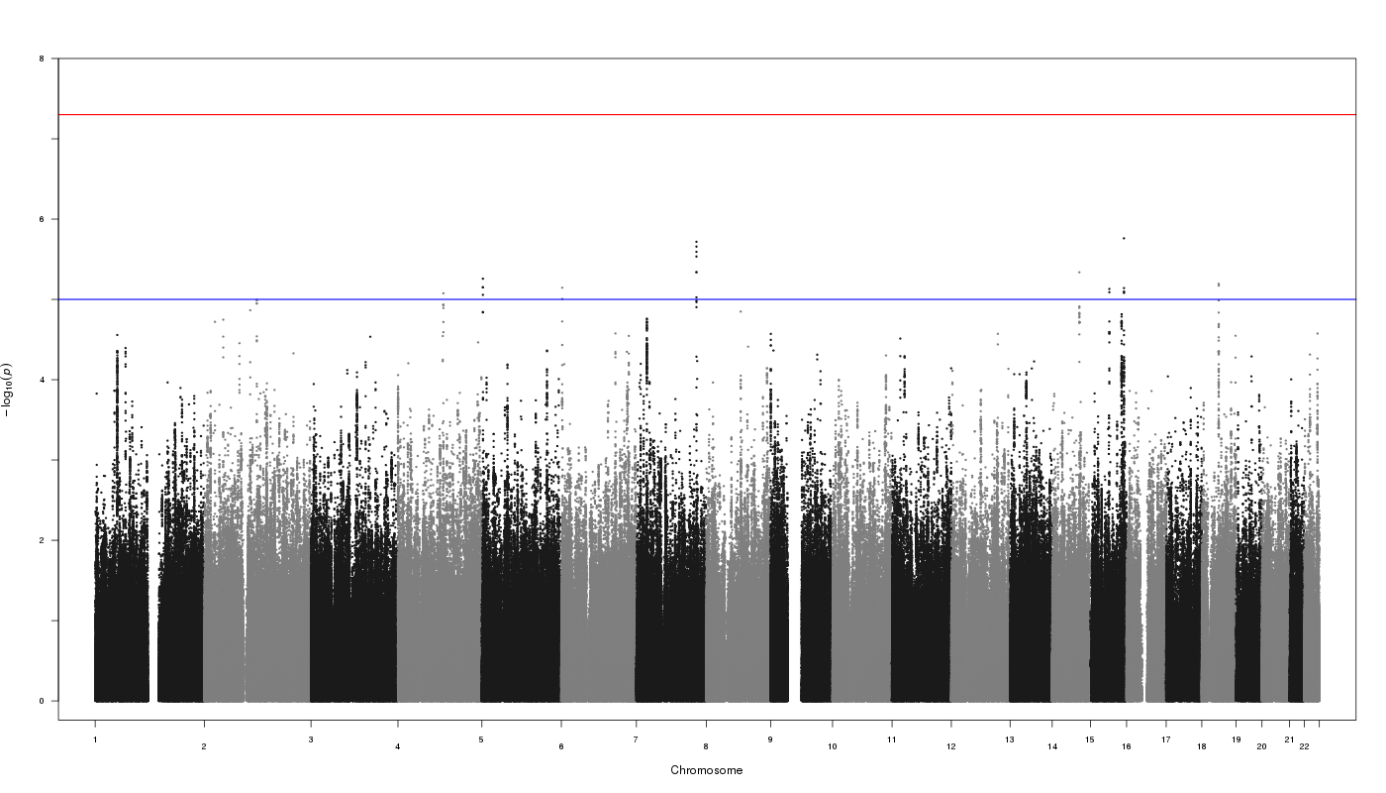


k. Queensland Institute of Medical Research Berghofer adults (QIMR Berghofer adults)


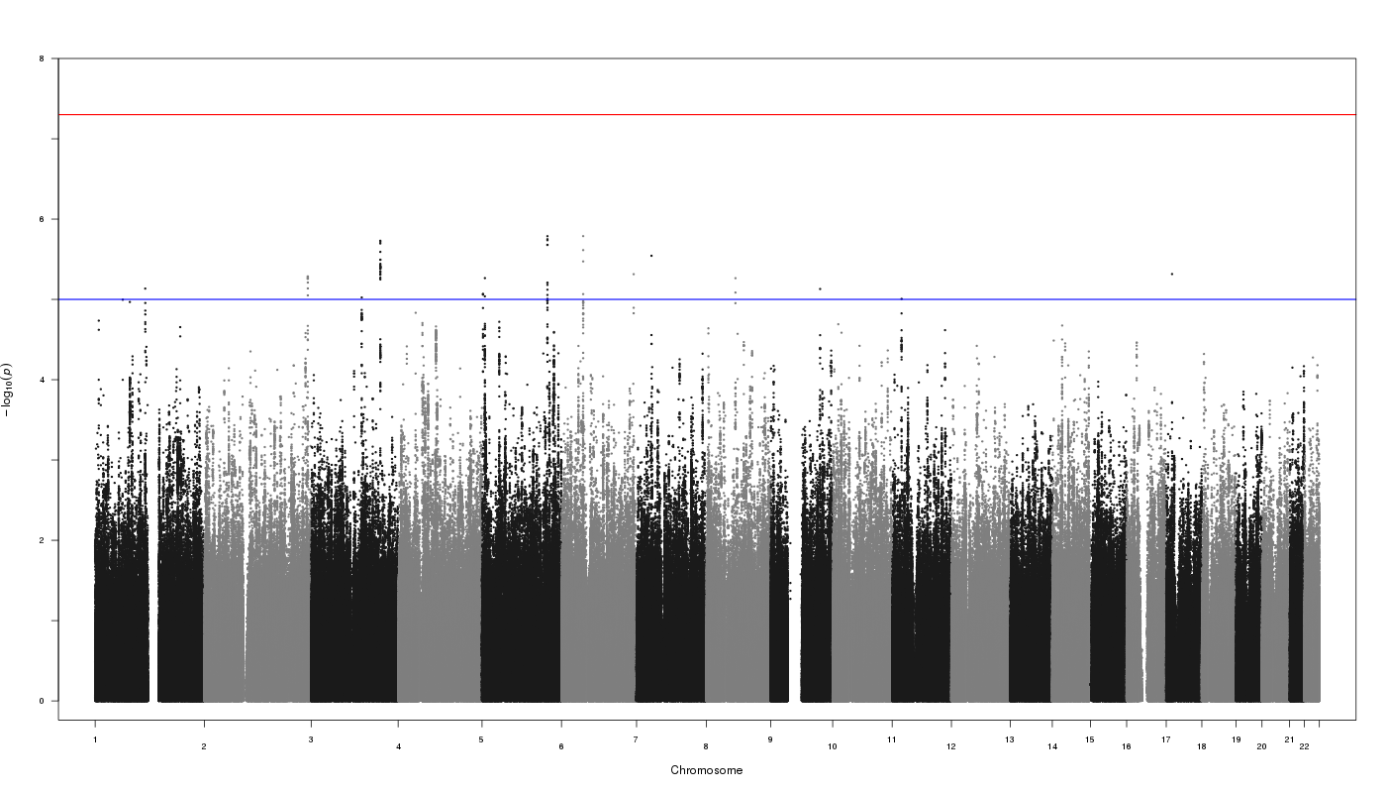


l. Tracking Adolescents’Individual Lives Survey (TRAILS)


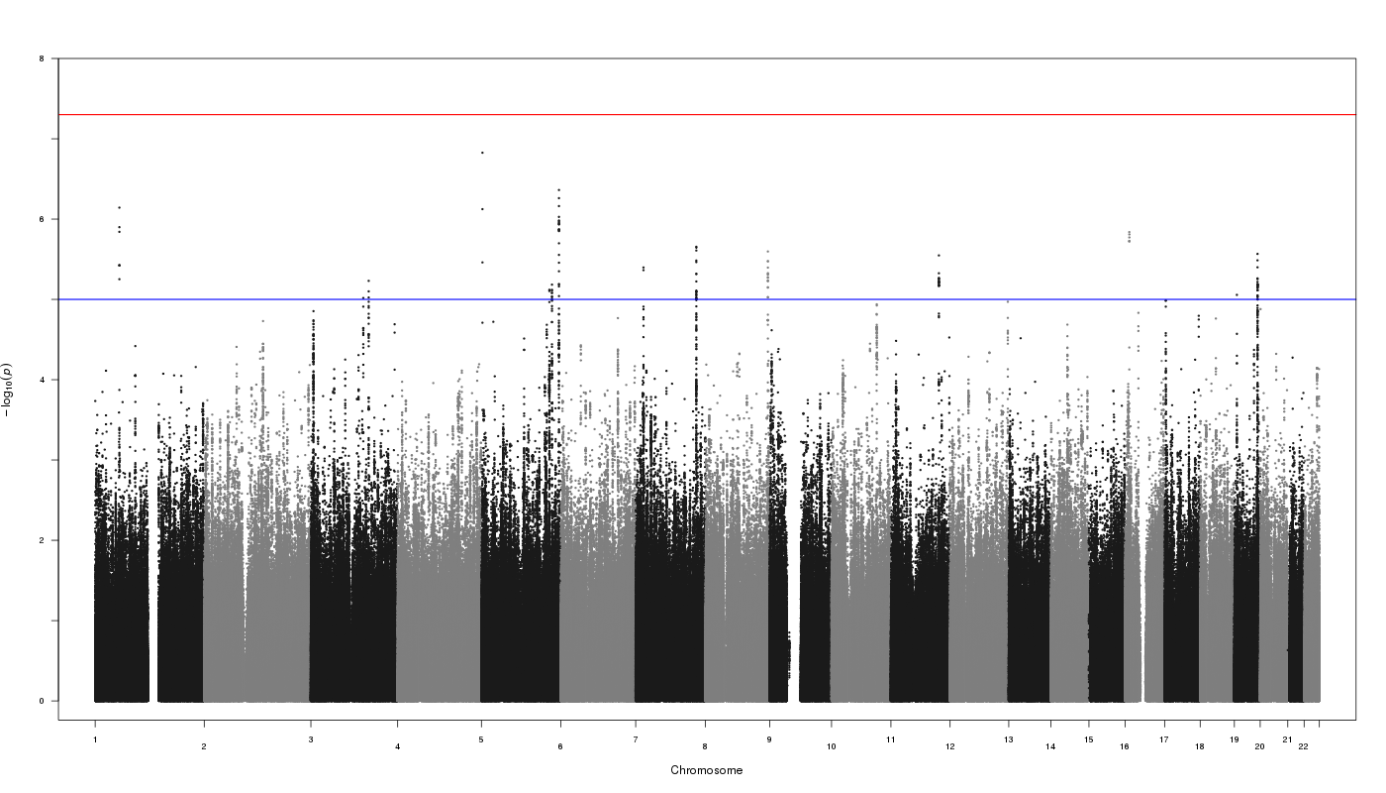


m. Utrecht **
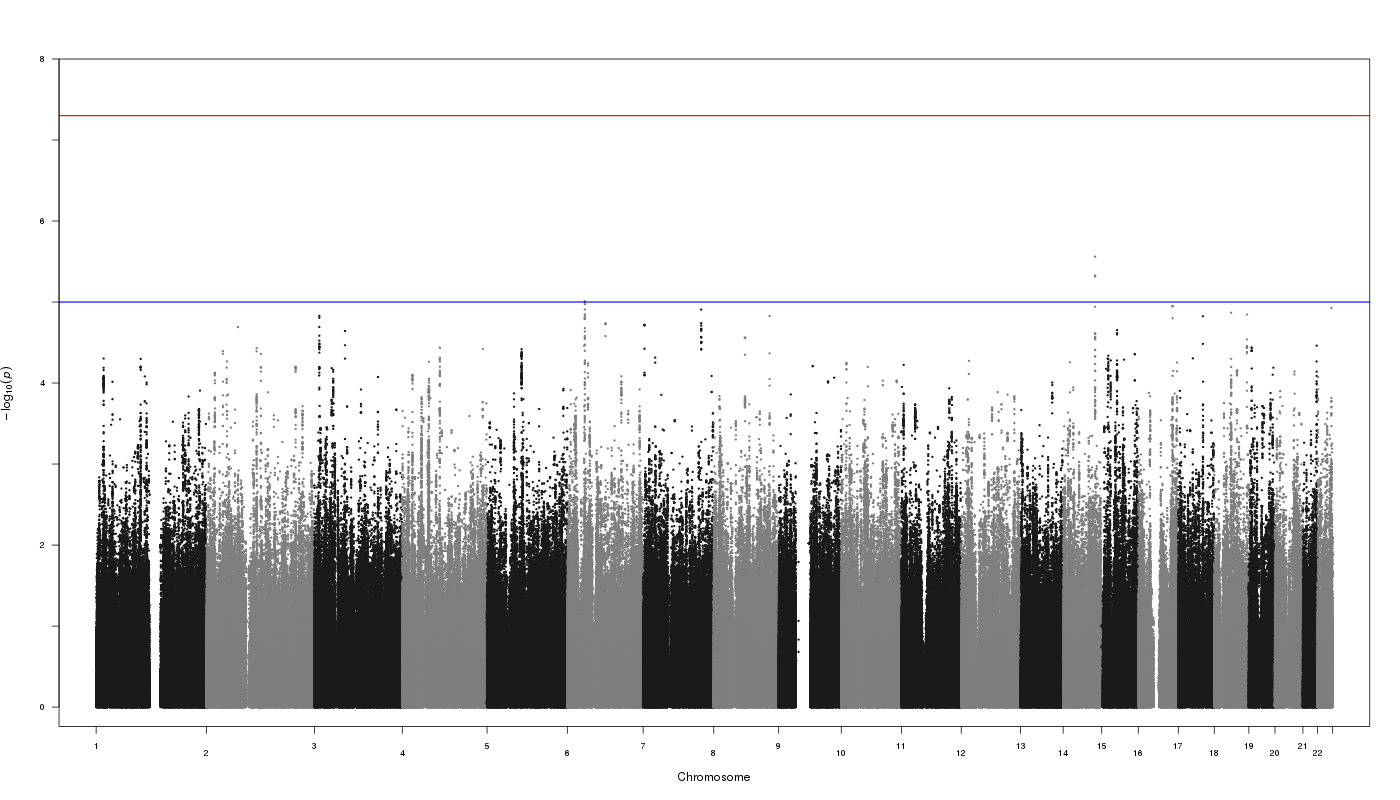
**

# Figure S3a-m. Quantile-quantile plots for lifetime cannabis use per sample

a. Avon Longitudinal Study of Parents and Children (ALSPAC) – *lambda=1.01*


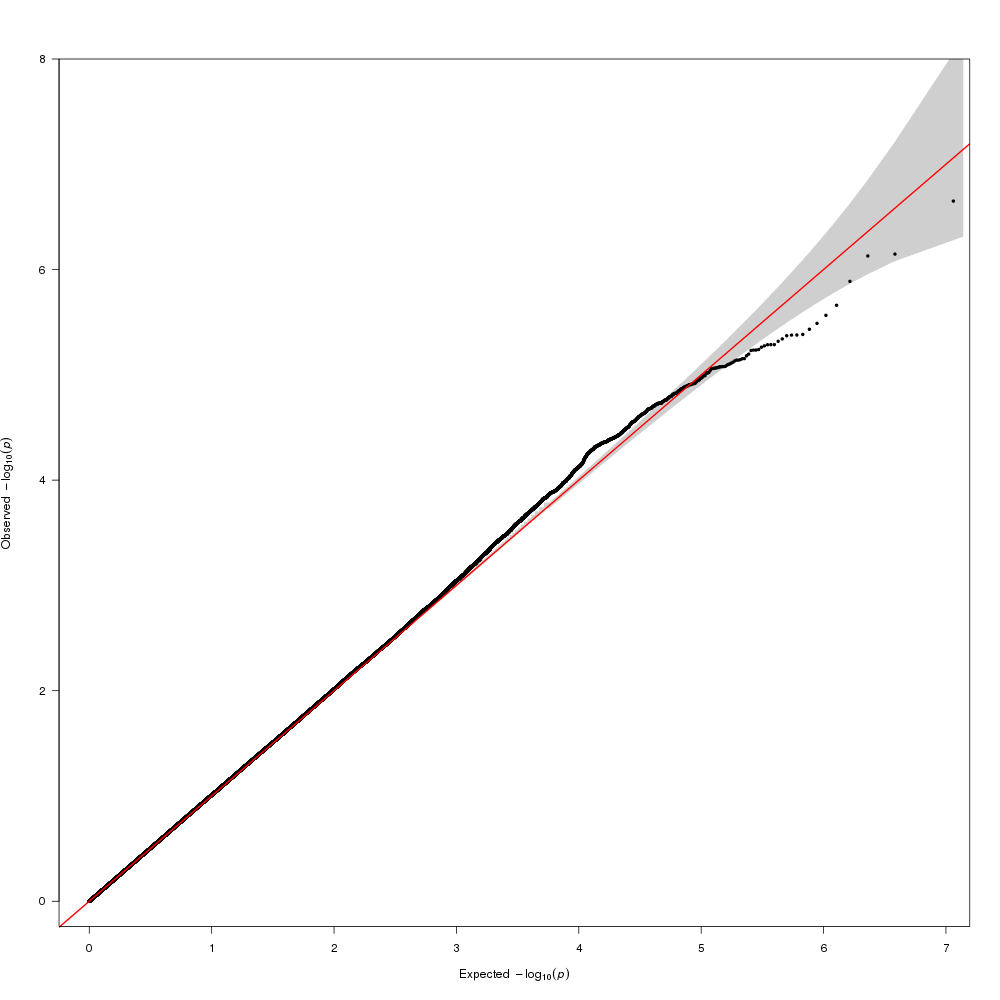


b. Brisbane Longitudinal Twin Study (BLTS) – *lambda=1.02*.


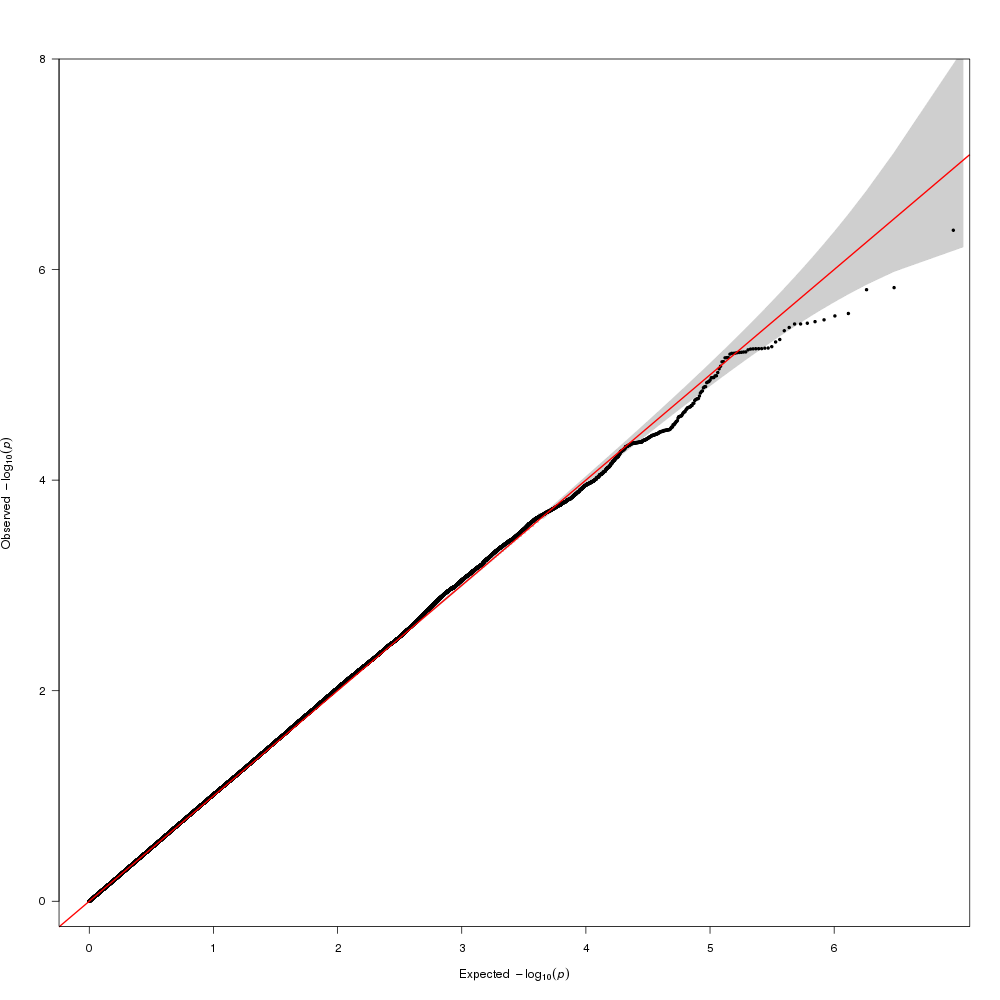


c. Center for Antisocial Drug Dependence (CADD) – *lambda=1.00.*


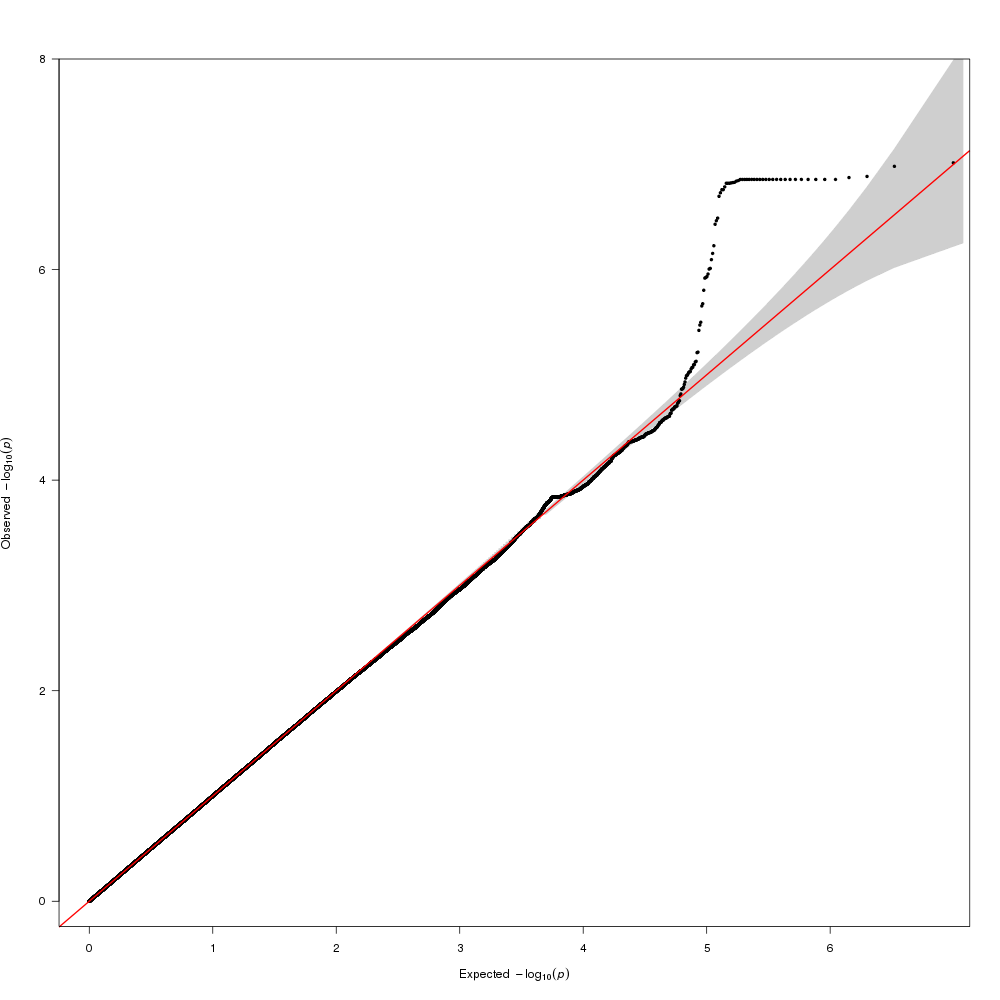


d. Estonian Genome Center University of Tartu -1 (EGCUT1) – *lambda=1.04.*


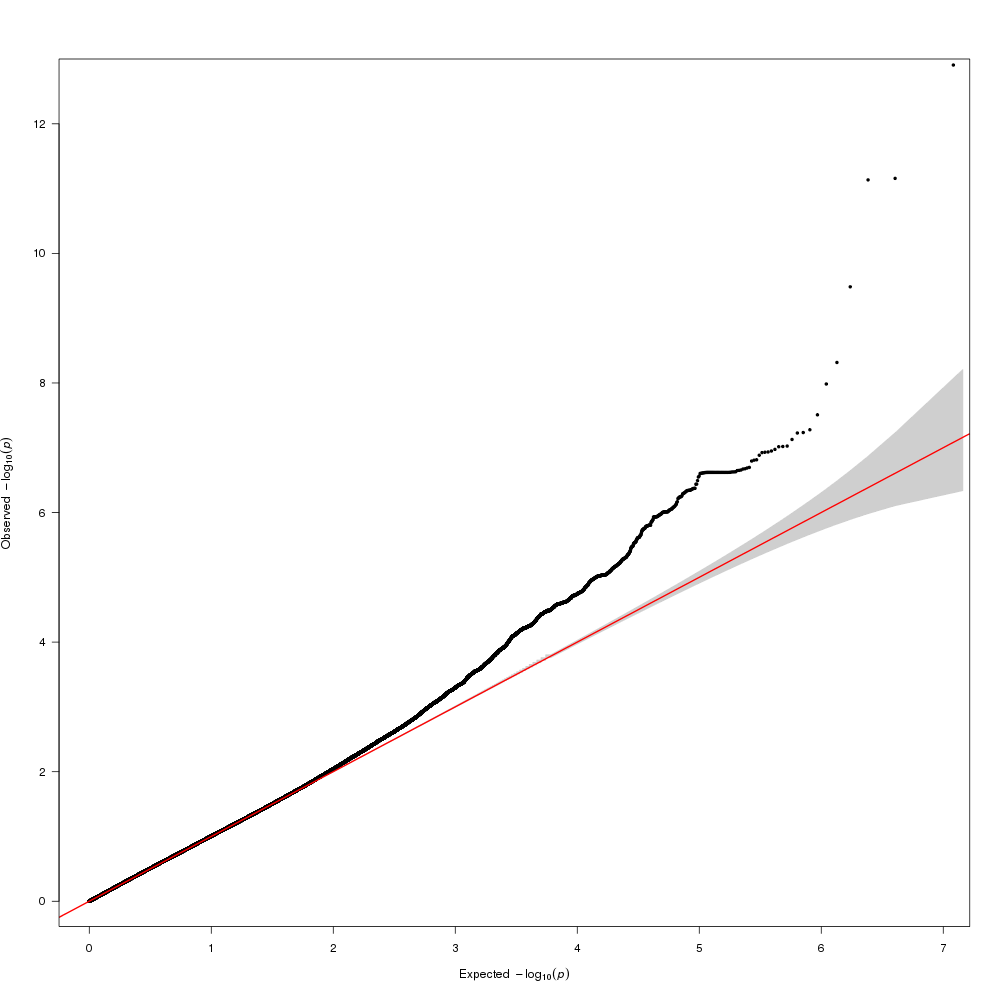


e. Estonian Genome Center University of Tartu -2 (EGCUT2) – *lambda=1.03.*


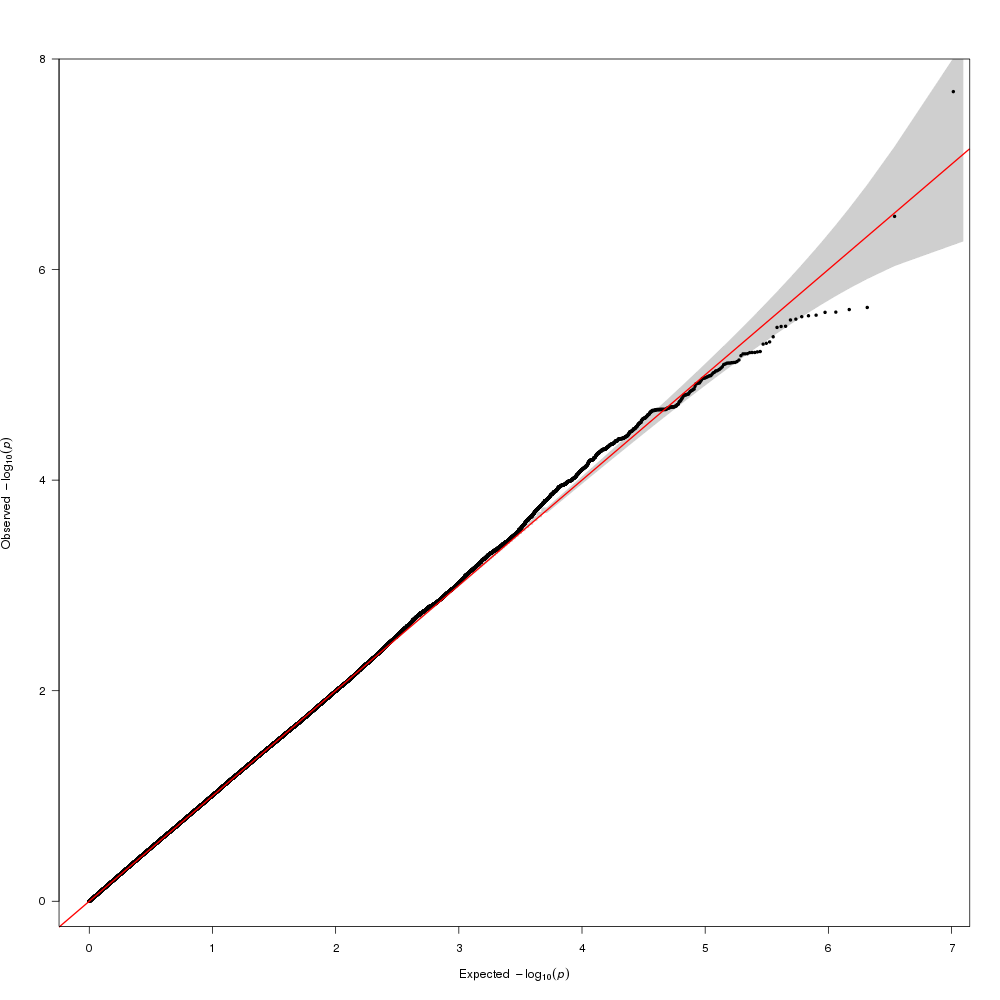


f. Finnish Twin Cohort (FinnTwin12 & FinnTwin16) – *lambda=1.02.*


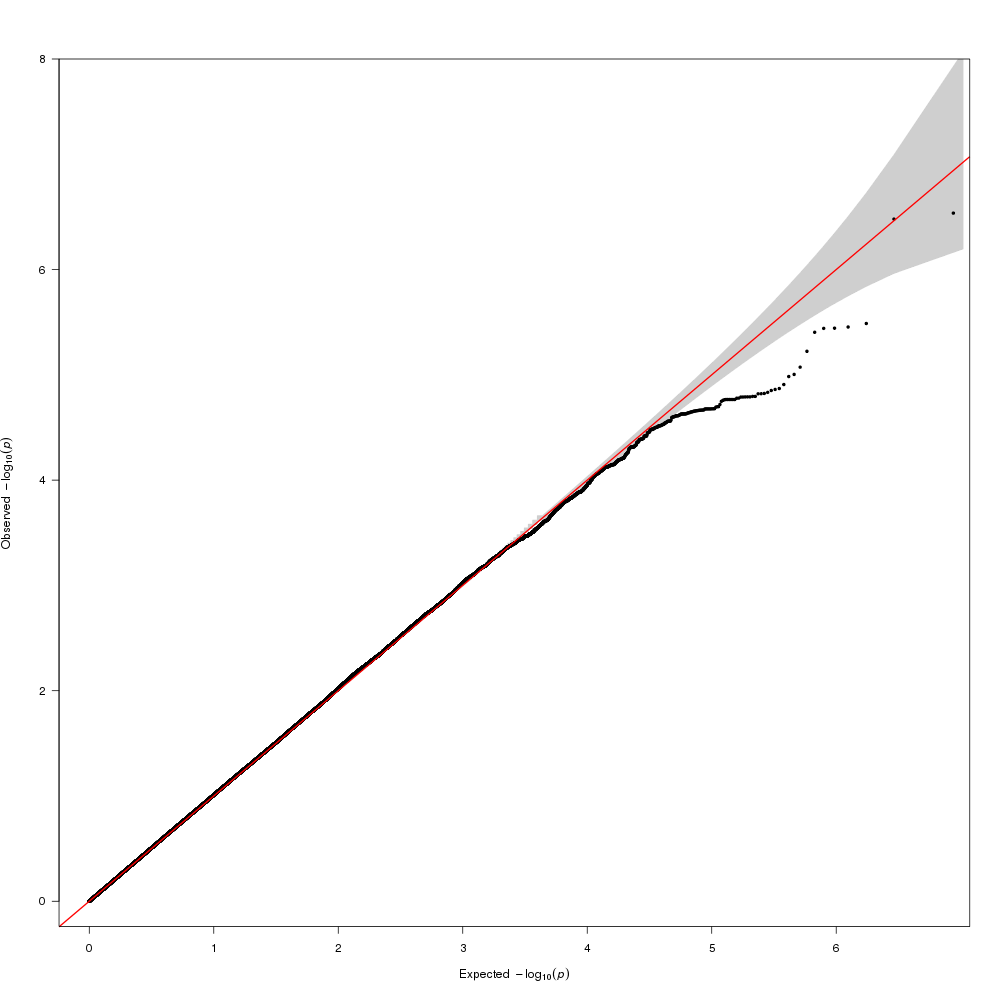


g. Genetics of Substance Dependence - European American (Yale Penn EA) – *lambda=1.00.*


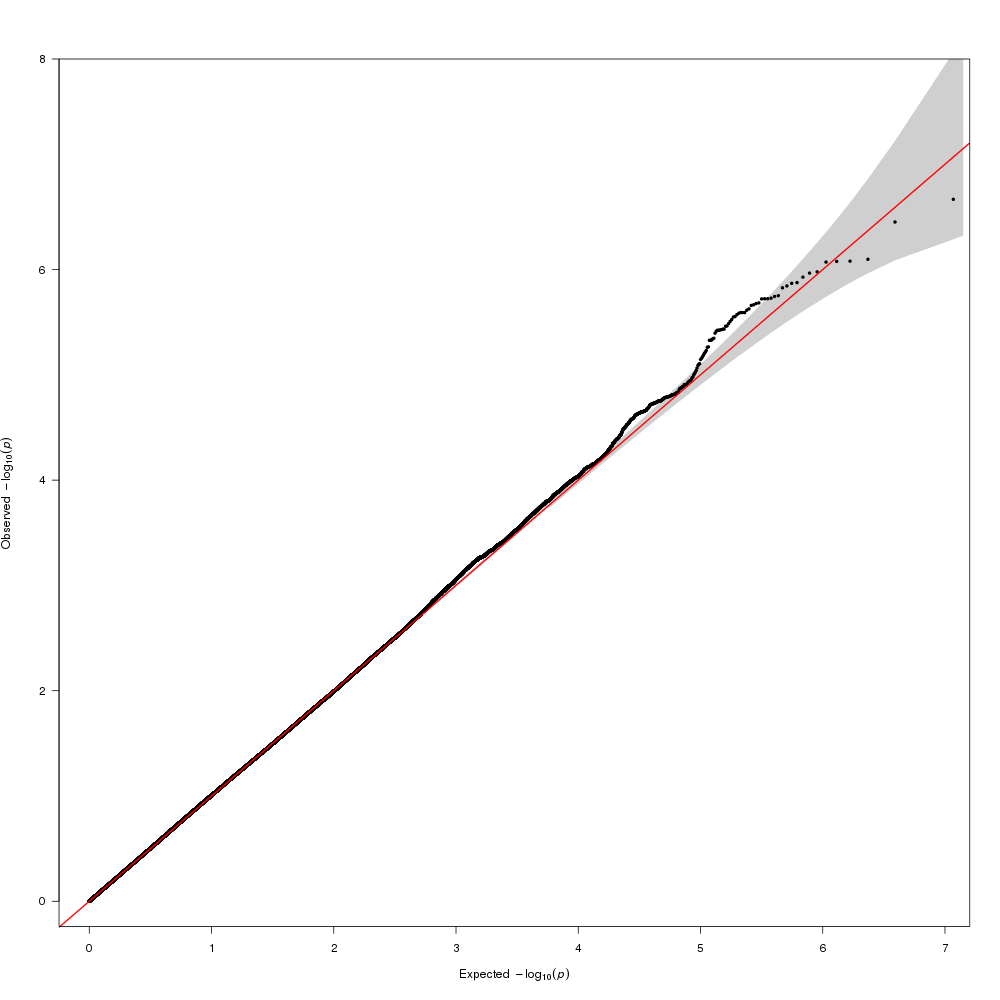


h. Hospital Universitari Vall d'Hebron – Barcelona (HUVH-Barcelona) –*lambda=1.04.*


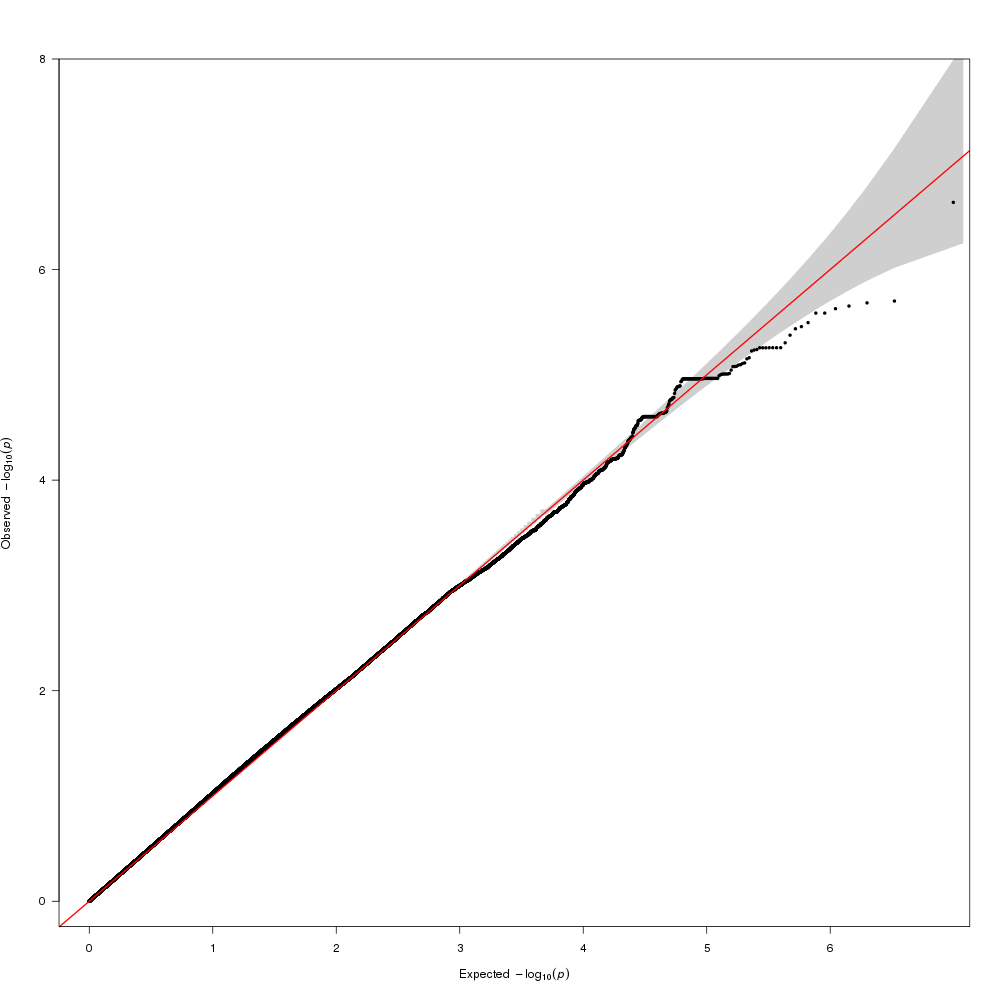


i. Minnesota Center for Twin and Family Research (MCTFR) – *lambda=1.02*


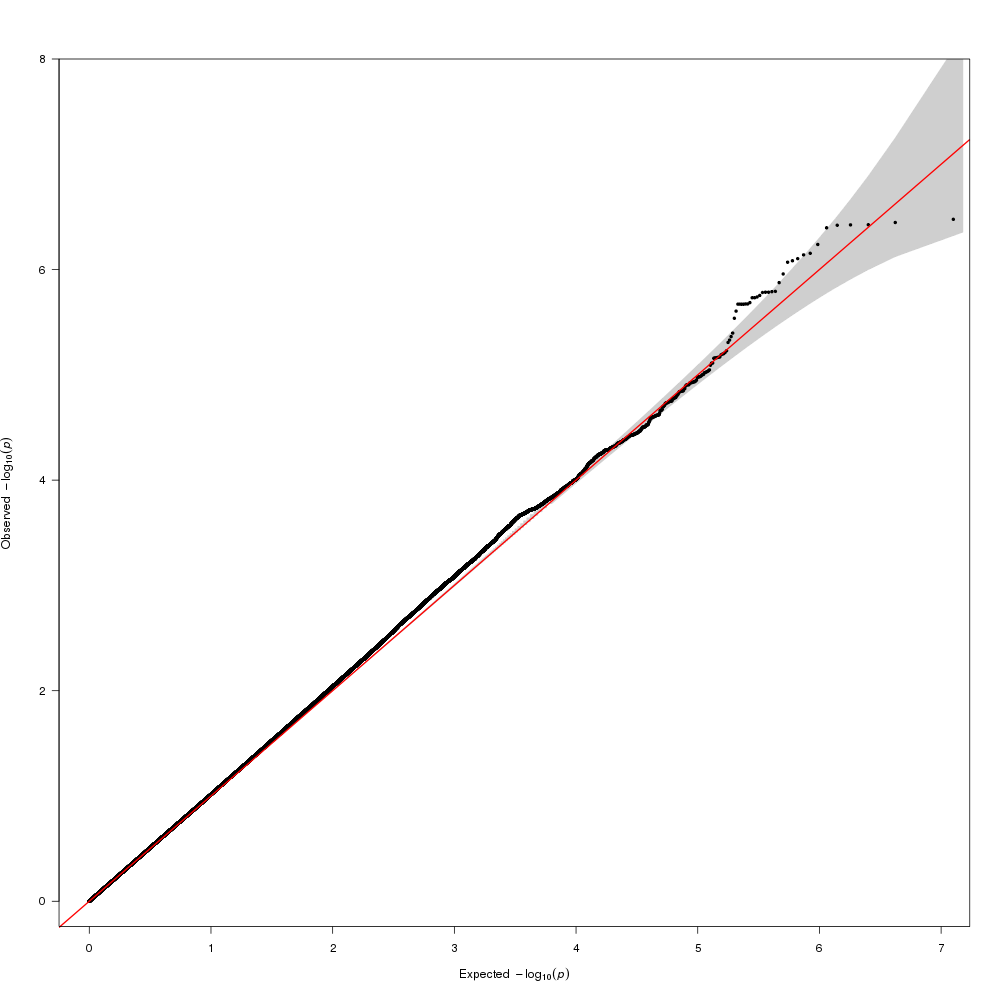


j. Netherlands Twin Register (NTR) – *lambda=1.01.*


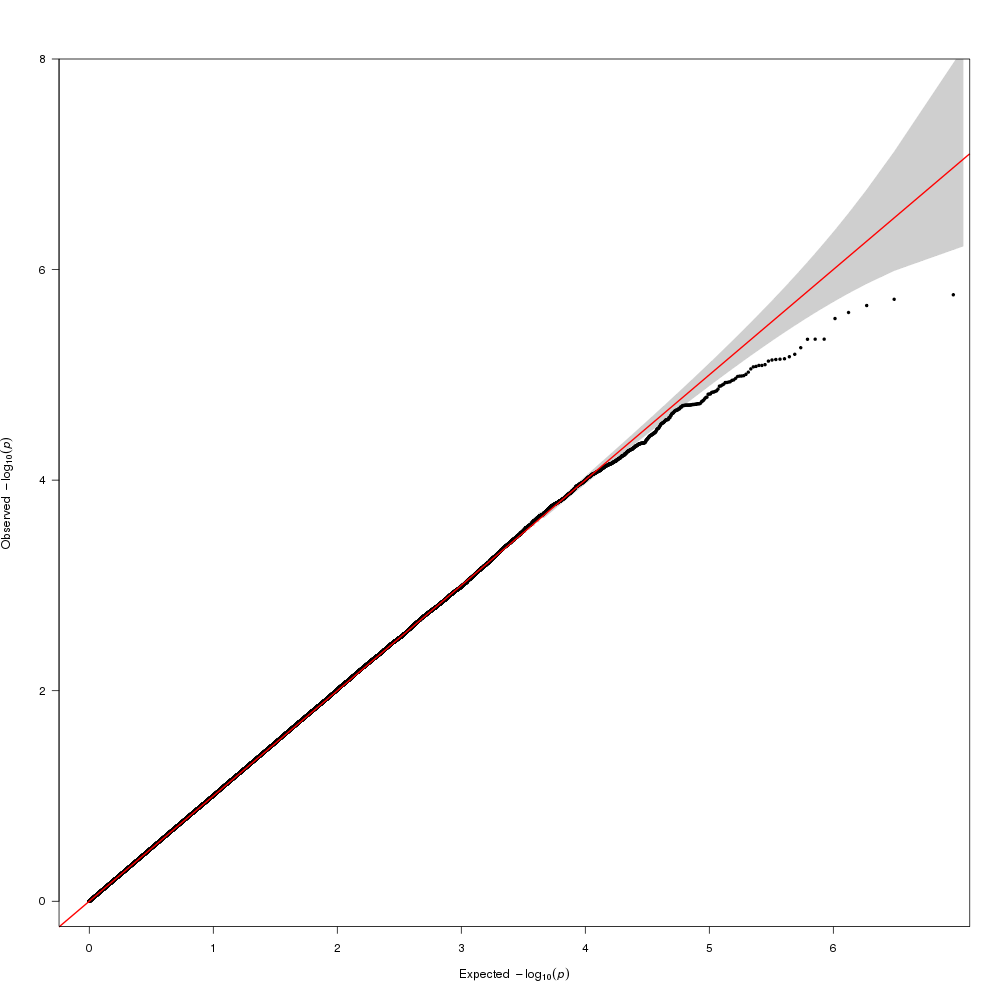


k. Queensland Institute of Medical Research Berghofer adults (QIMR Berghofer adults) – *lambda=1.03.*


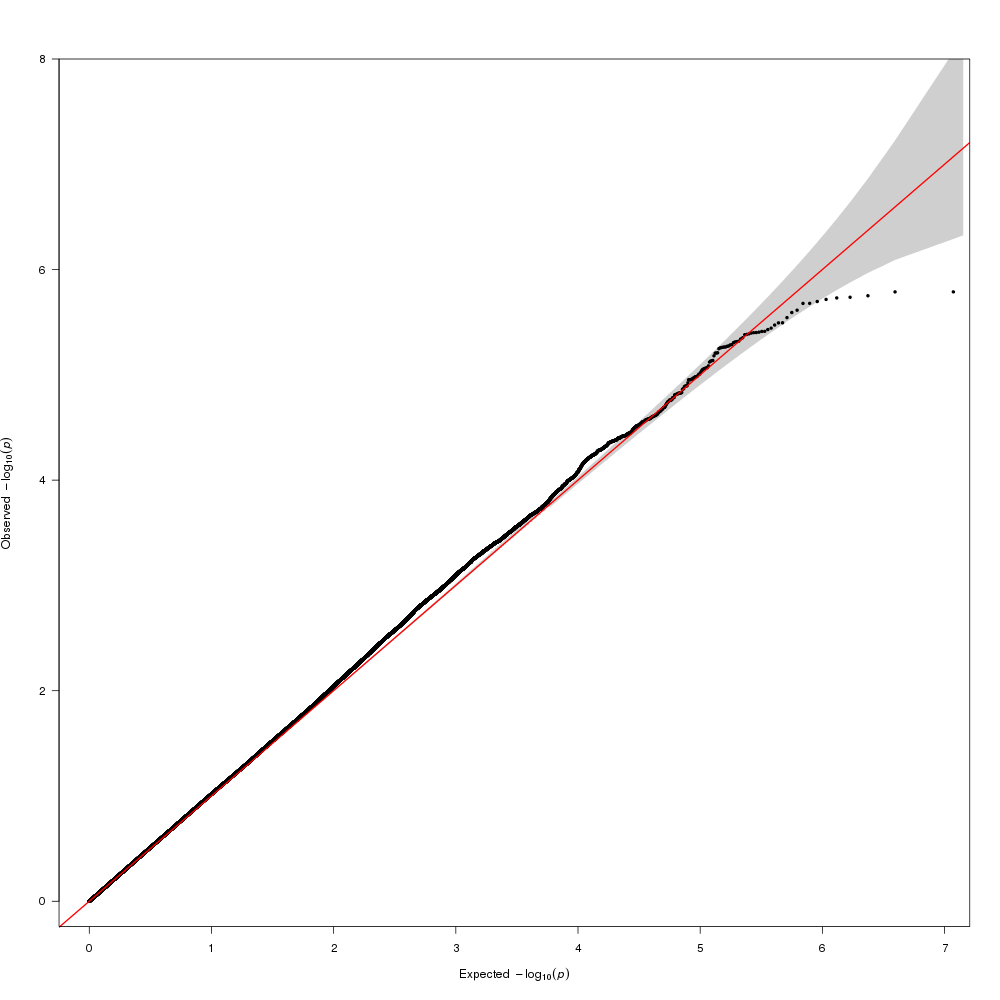


l. Tracking Adolescents’Individual Lives Survey (TRAILS) – *lambda=1.01.*


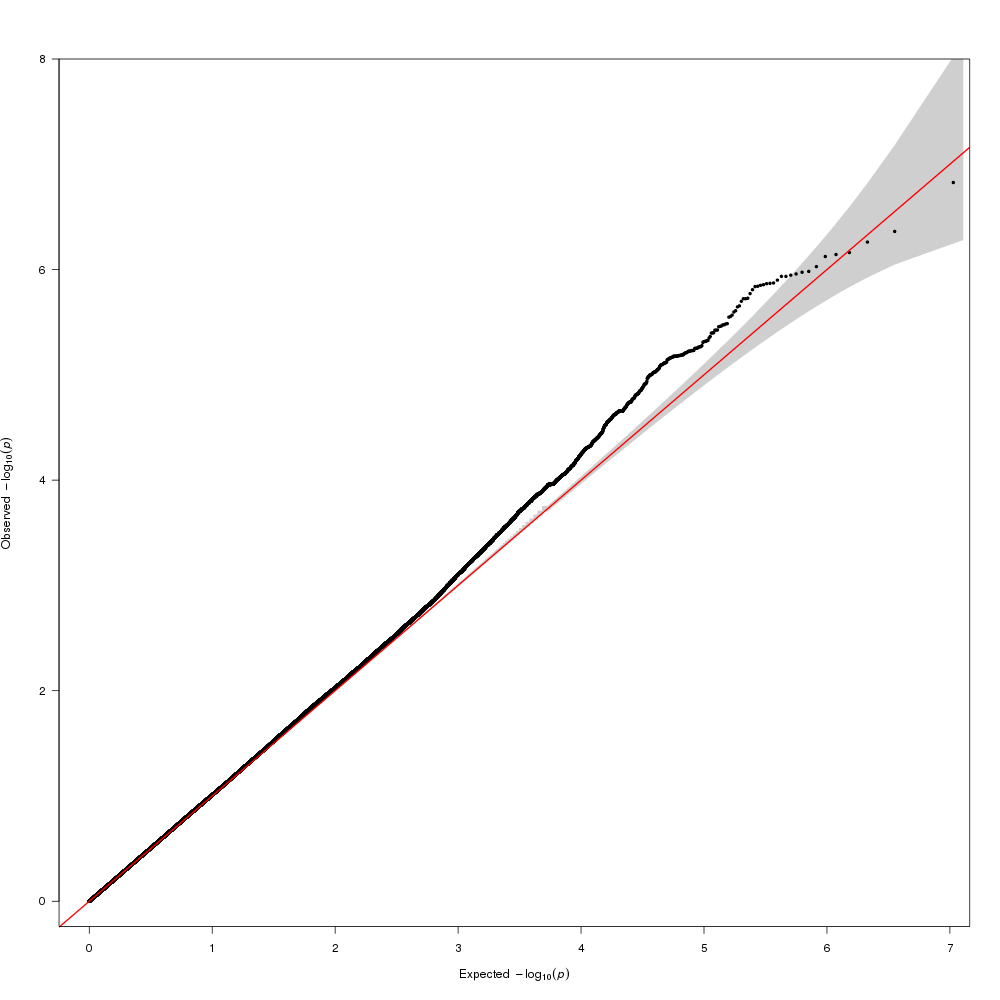


m. Utrecht – ­*lambda=1.02*


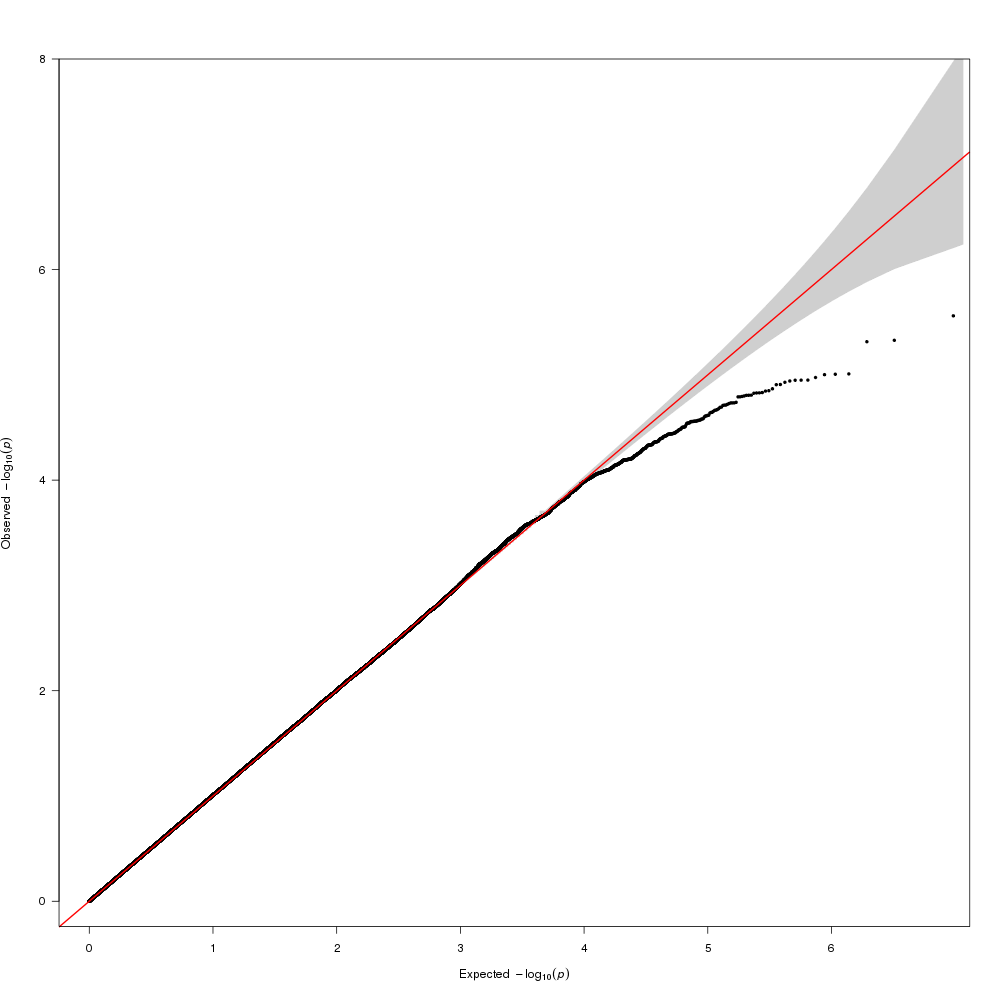


# Figure S4a-j. Regional association plot showing signal around top SNPs

Regional association plots corresponding to the meta-analysis results for the top 10 loci. (a–j) For each plot, the −log10 *P* values (*y* axis) of the top significant SNPs are shown according to their chromosomal positions (*x* axis). The loci are at 15q26.1 (a), 12p11.22 (b), 4q31.1 (c), 11q23.2 (d), 11p15.5 (e), 2p16.3 (f), 2q31.1 (g), 3p21.31 (h), 5q12.1 (i) and 1q31.3 (j). The estimated recombination rates from the 1000 Genomes Project March 2012 release are shown as blue lines, and the genomic locations of genes within the regions of interest in the NCBI Build 37 human assembly are shown as arrows. SNP color represents LD with the most highly associated SNP at each locus.

a. Region 15q26.2

b. Region 12p11.22

c. Region 4q31.1

d. Region 11q23.2

e. Region 11p15.5

f. Region 2p16.3

g. Region 2q31.1

h. Region 3p21.31

i. Region 5q12.1

j. Region 1q31.3

# Figure S5. Forest plots key SNPs in the 5 significant regions


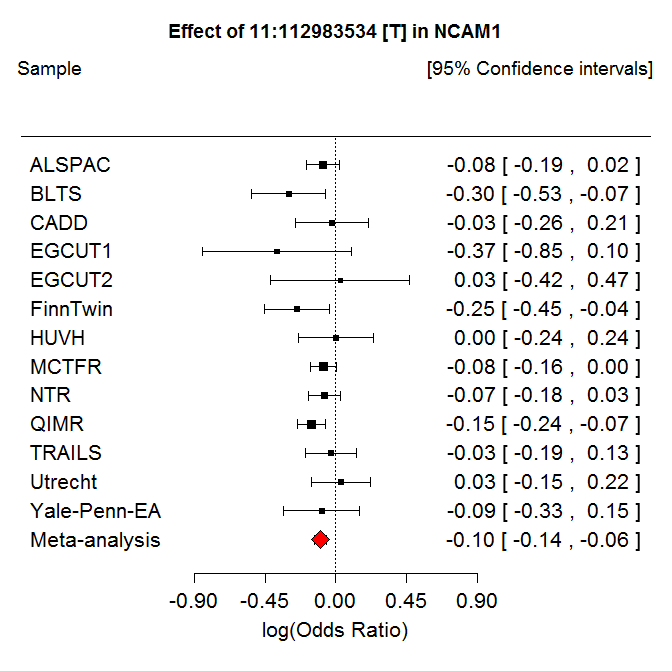


**
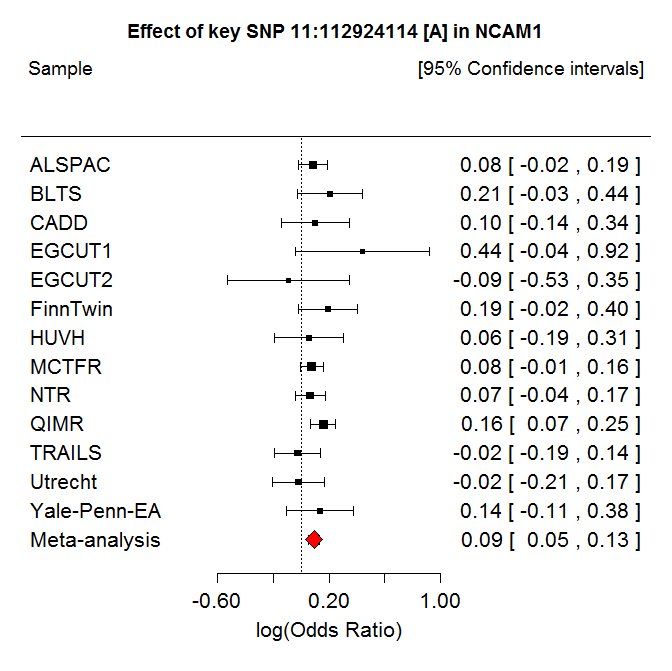
**

**
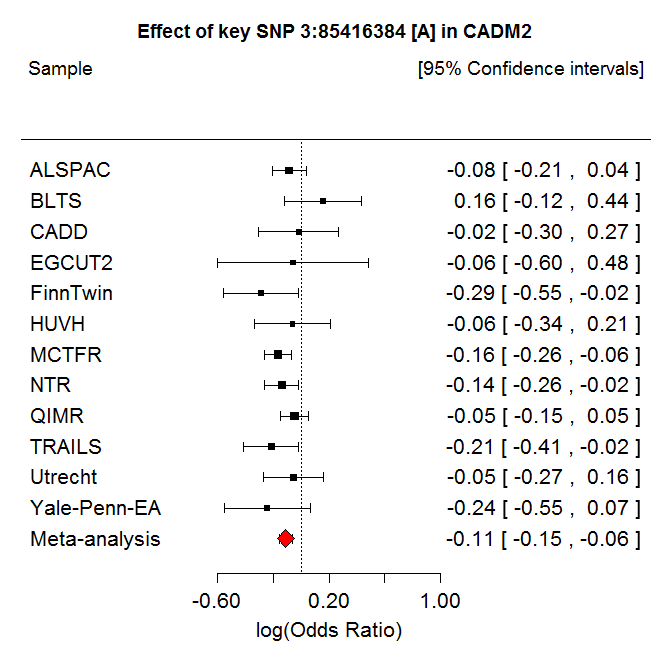
**

**
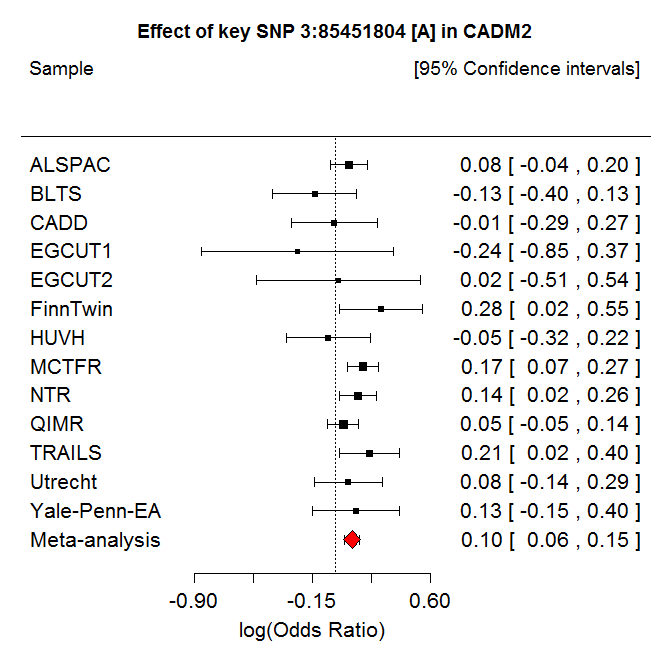
**

**
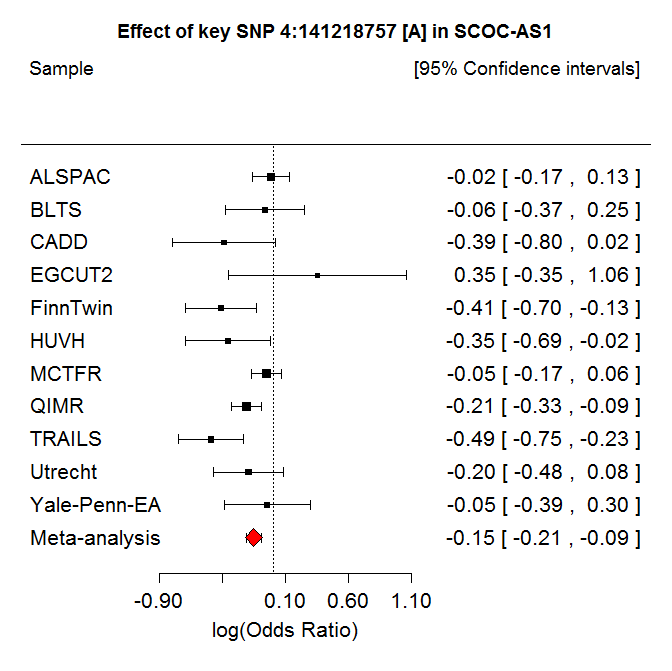
**

**
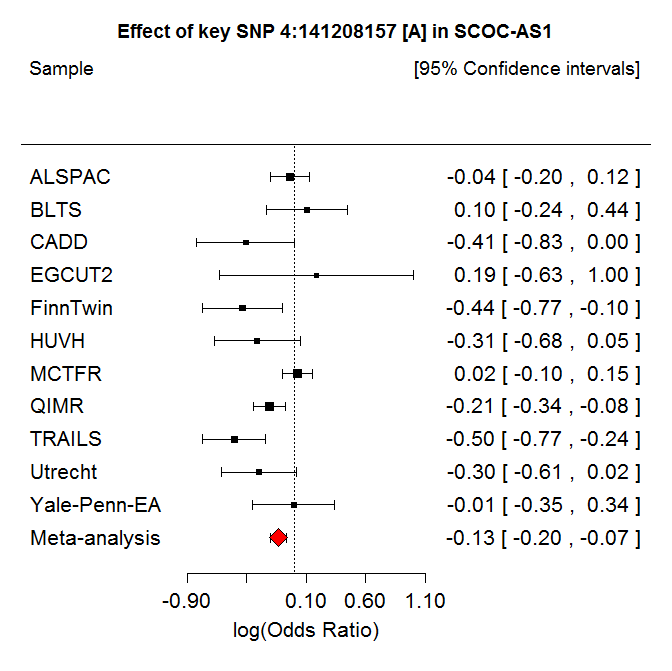
**

**
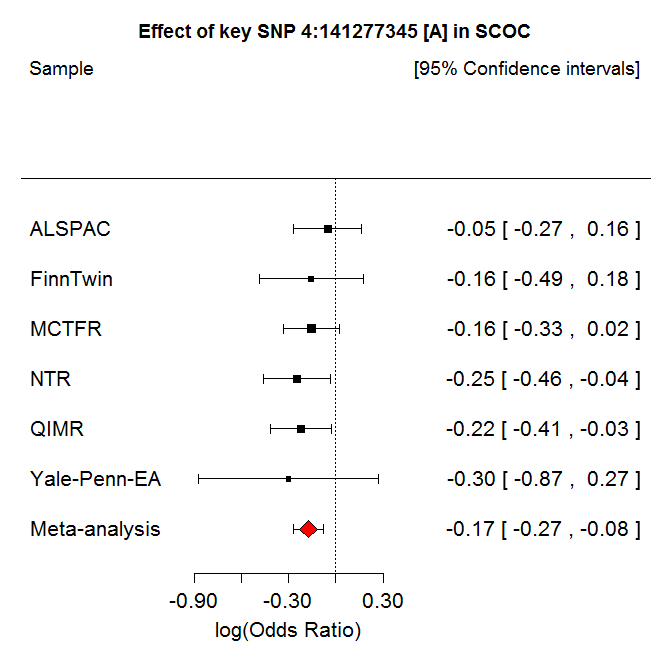
**

**
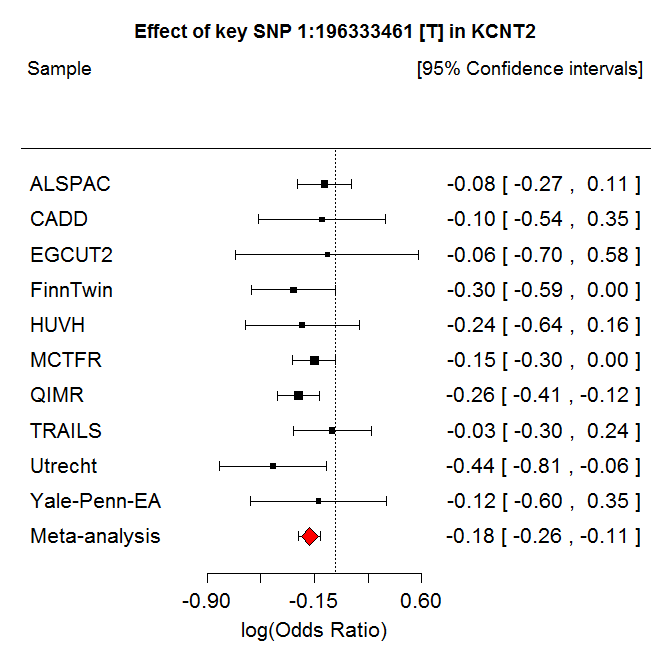
**

**
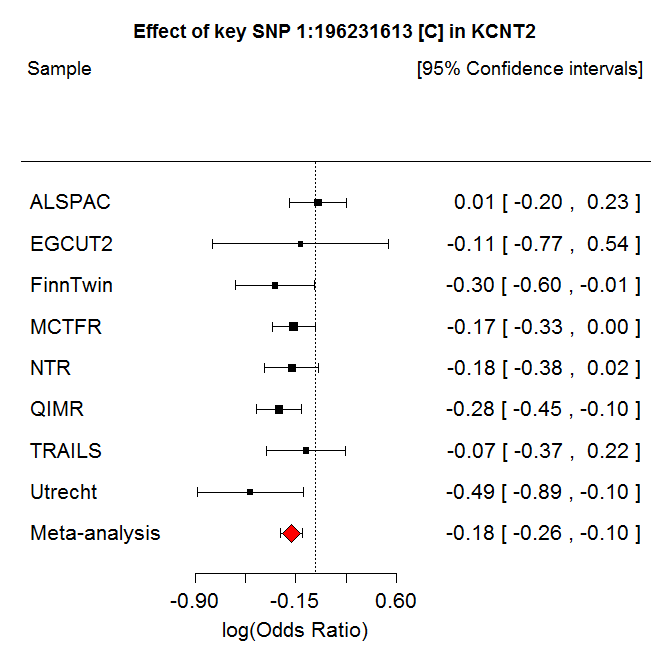
**

# Figure S6a-e. Regional plots around the 5 significant regions

Regional association plots corresponding to the gene-based association results for the top 5 genes/genetic regions. (a–j) For each plot, the −log10 *P* values (*y* axis) of the top significant SNPs are shown according to their chromosomal positions (*x* axis).

a. NCAM1


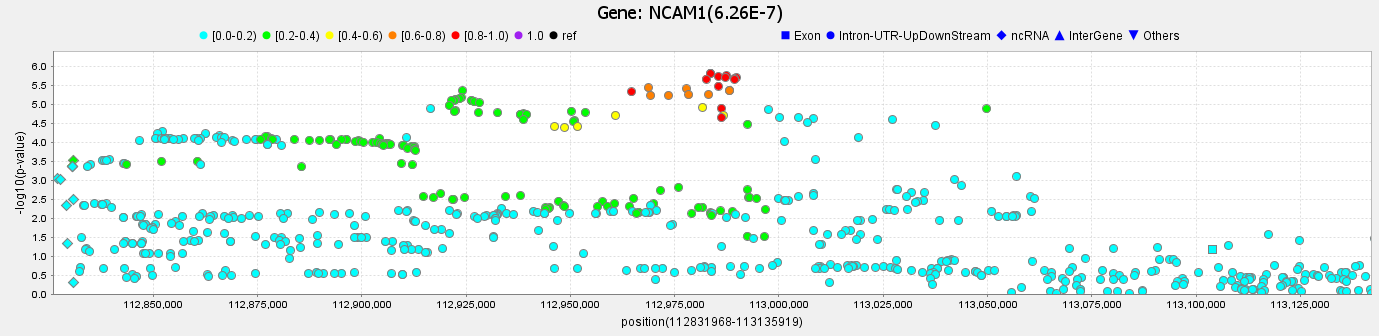


b. CADM2


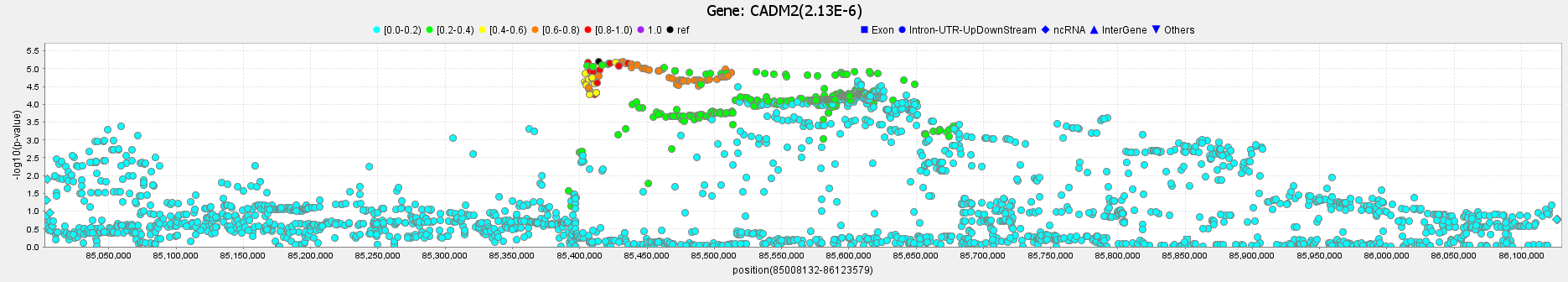


c. SCOC-AS1


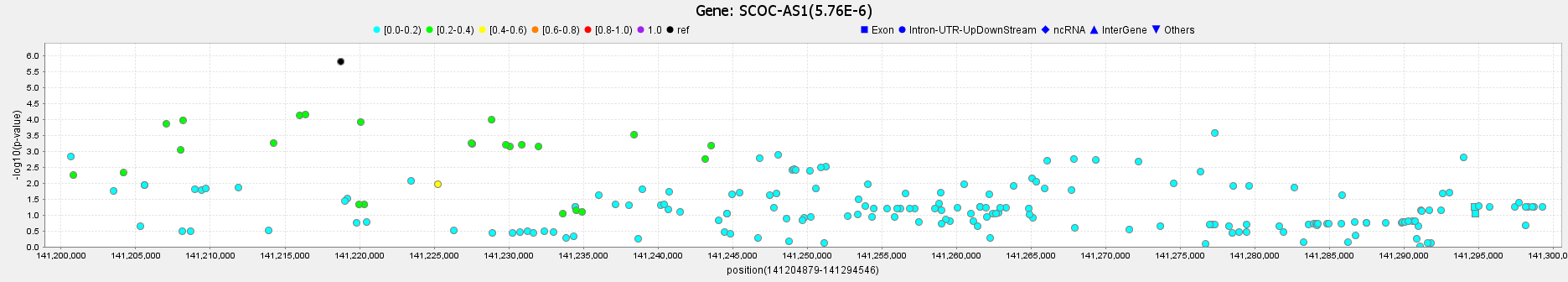


d. SCOC


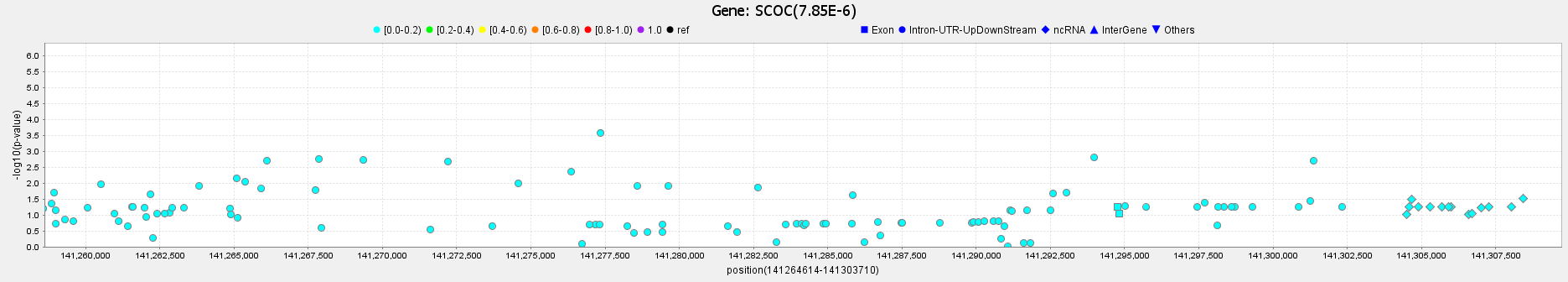


e. KCNT2


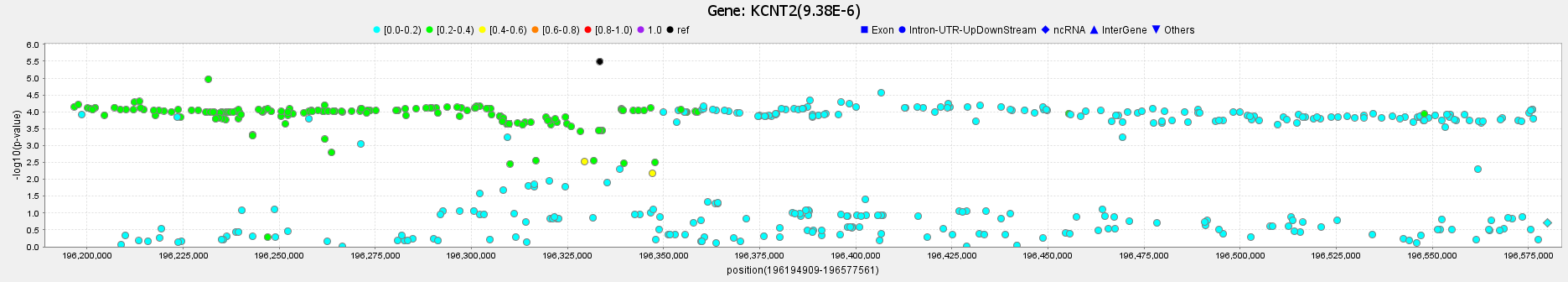

Supplement: Supplementary Information [file tp201636x10.docx]
